# Information-Theoretic Reliability Analysis of Consecutive *r*-out-of-*n*:G Systems via Residual Extropy

**DOI:** 10.3390/e27111090

**Published:** 2025-10-22

**Authors:** Anfal A. Alqefari, Ghadah Alomani, Faten Alrewely, Mohamed Kayid

**Affiliations:** 1 Department of Statistics and Operations Research, College of Science, Qassim University, P.O. Box 6644, Buraydah 51482, Saudi Arabia; aa.alqefari@qu.edu.sa; 2 Department of Mathematical Sciences, College of Science, Princess Nourah Bint Abdulrahman University, P.O. Box 84428, Riyadh 11671, Saudi Arabia; gaalomani@pnu.edu.sa; 3 Department of Mathematics, College of Science, Jouf University, P.O. Box 2014, Sakaka 72388, Saudi Arabia; 4 Department of Statistics and Operations Research, College of Science, King Saud University, P.O. Box 2455, Riyadh 11451, Saudi Arabia; drkayid@ksu.edu.sa

**Keywords:** residual extropy, reliability inference, consecutive *r*-out-of-*n*:G systems, information-theoretic measures, stochastic ordering, aging classes (IFR/DFR), reliability bounds, 62N05, 94A17

## Abstract

This paper develops an information-theoretic reliability inference framework for consecutive r-out-of-n:G systems by employing the concept of residual extropy, a dual measure to entropy. Explicit analytical representations are established in tractable cases, while novel bounds are derived for more complex lifetime models, providing effective tools when closed-form expressions are unavailable. Preservation properties under classical stochastic orders and aging notions are examined, together with monotonicity and characterization results that offer deeper insights into system uncertainty. A conditional formulation, in which all components are assumed operational at a given time, is also investigated, yielding new theoretical findings. From an inferential perspective, we propose a maximum likelihood estimator of residual extropy under exponential lifetimes, supported by simulation studies and real-world reliability data. These contributions highlight residual extropy as a powerful information-theoretic tool for modeling, estimation, and decision-making in multicomponent reliability systems, thereby aligning with the objectives of statistical inference through entropy-like measures.

## 1. Introduction

Information-theoretic measures, particularly entropy and its variants, have recently emerged as powerful tools in reliability inference and statistical modeling, providing new perspectives on uncertainty quantification, system characterization, and inferential methodologies. Within this framework, residual extropy offers a complementary dual to entropy, enabling innovative approaches to the study of consecutive *r*-out-of-*n*:G systems, which represent a fundamental class of multicomponent reliability structures. While much of the existing literature has focused on the information properties of general technical systems, growing attention has turned toward consecutive *r*-out-of-*n* configurations and their variants, motivated by their wide range of engineering applications. Such systems are typically classified as linear or circular, depending on the spatial arrangement of their components, and as good or failure systems according to their operating criteria. In particular, a linear consecutive *r*-out-of-*n*:G system consists of *n* independent and identically distributed components arranged sequentially, with system performance determined by the operation of *r* contiguous components. For illustration, consider a street with *n* parallel parking spaces designed for standard-sized vehicles; a bus, due to its larger dimensions, requires two contiguous spaces. The parking system is operational if at least two adjacent spaces are available, which corresponds to a linear consecutive 2-out-of-*n*:G system (see Gera [1]). In particular, the consecutive system framework includes the series system as the case r=n and the parallel system as the case r = 1. These structural variations highlight the versatility of consecutive systems and their importance in practical applications, where they serve as effective models for diverse reliability scenarios. Consequently, the reliability properties of consecutive systems have been widely studied under various assumptions, with significant contributions from several authors [2,3,4,5,6,7,8]. More recently, Eryilmaz [9] showed that the lifetime distribution of linear consecutive r-out-of-n:G systems admits a simple and tractable form when 2r≥n. Motivated by this simplification, the present study focuses on scenarios where this condition holds, as it facilitates both the mathematical analysis and the derivation of useful results.

Assume that the lifetime of each component in these systems is represented by $T_{1},T_{2},\ldots ,T_{n}$, where the corresponding order statistics are denoted by $T_{1:n},T_{2:n},\ldots ,T_{n:n}$. We assume that the lifetimes of the components follow a probability density function (pdf) h(x) and a cumulative distribution function (cdf) H(x). The lifetime of the system is denoted by $T_{r|n:G}$. Thus, if 2r≥n, the reliability (survival) function of $T_{r|n:G}$ is given by the following (see, e.g., Eryilmaz [9]):

(1) $$\begin{array}{c} {\bar{H}}_{r|n:G}(x)=(n-r+1){\bar{H}}^{r}(x)-(n-r){\bar{H}}^{r+1}(x),x>0, \end{array}$$

where $\bar{H}(x)=P(T>x)$, is the reliability function of the component lifetimes. It follows that:

(2) $$\begin{array}{c} h_{r|n:G}(x)=r(n-r+1){\bar{H}}^{r-1}(x)h(x)-(r+1)(n-r){\bar{H}}^{r}(x)h(x),\;x>0. \end{array}$$

Ebrahimi et al. [10] significantly advanced the link between information theory and reliability by examining the information properties of order statistics. Building on Shannon’s seminal work [11], differential entropy has since become a fundamental concept in probability theory and one of the most widely used measures of uncertainty.

Let T be a nonnegative random variable with the pdf h(⋅). It is known that the Shannon differential entropy of T is expressed as H(T)=−E[logh(T)], provided that the expectation exists. Lad et al. [12] recently introduced extropy, a novel measure of uncertainty dual to entropy. Thus, extropy of a nonnegative random variable T supported on the interval [0,∞) with pdf h and cdf H is defined as follows:

(3) $$\begin{array}{ll} R\left(T\right) & =-\frac{1}{2}\int_{0}^{\infty }\;\;h^{2}\left(x\right)dx\\ & =-\frac{1}{2}\int_{0}^{1}\;\;h\left(H^{-1}\left(u\right)\right)du,\;\;(\mathrm{taking}\;u=H(x))\\ & =-\frac{1}{2}E\left[h\left(H^{-1}\left(U\right)\right)\right], \end{array}$$

where $H^{-1}(u)=inf\{x;H(x)\geq u\}$, for u∈[0,1], denotes the quantile function or left continuous inverse of H and the random variable U is uniformly distributed on [0, 1]. Like entropy, as R(T) increases, the h(x) approaches to uniformity, indicating that it evaluates the uniformity of the distribution. As the probability density function becomes less concentrated, predicting the outcome of a random draw from *h*(*x*) becomes more difficult. Distributions with sharp peaks are associated with low extropy, whereas those with more evenly spread probabilities correspond to higher extropy.

Although entropy H(f) and extropy J(f) serve as measures of uncertainty and dispersion, they exhibit important differences that prevent them from inducing the same ordering over distributions. One key distinction lies in their ranges: differential entropy can take any real value [−∞, ∞], whereas extropy is always non-positive, taking values in [−∞,0). Moreover, for any continuous distribution with density f, it holds that J(f) < H(f), a consequence of the inequality $2x\log x<x^{2}$ for all x>0. Additionally, if $\sigma^{2}\left(X\right)=E{\left(X-\mu \right)}^{2}$, denotes the variance of *X*, then $\sigma^{2}(X)<\infty$ implies E(|X|)<∞, ensuring $J\left(f\right)<\infty$. This is due to the fact that $J\left(f\;\right)<\;H\left(f\;\right)<E(|X|)<\infty$, but the converse is not true.

A notable advantage of extropy is its computational tractability, especially for complex distributions such as mixture distributions. While entropy often involves intractable integrals for mixture models, extropy can frequently be expressed in closed form or computed more efficiently due to its quadratic structure in the probability density function. This makes extropy particularly useful in applications involving model comparison, clustering, or information-based inference with mixture densities. However, extropy also has limitations. Unlike entropy, which has deep connections to fundamental principles in physics (e.g., the second law of thermodynamics) and information theory (e.g., Shannon’s source coding theorem), extropy lacks such foundational interpretations. Additionally, because extropy is always bounded above by zero and behaves differently under transformations (e.g., scaling), it may be less intuitive or less directly applicable in certain theoretical contexts where entropy’s properties are essential.

In various situations, the engineers consider and quantify uncertainty in a system’s lifetime, as it directly impacts system reliability. Let T denote the lifetime of a new system. Let us assume that the uncertainty of the system is quantified by R(T). In certain scenarios, operators may possess information regarding the system’s current age. For instance, they may know that the system is operating at the time t and may be interested in measuring the uncertainty of its residual lifetime, i.e., $T_{t}=[T-t|T>t]$. In these situations, R(T) is no longer useful. Consequently, the residual extropy is introduced by (see, e.g., Toomaj et al. [13])

(4) $$R(T;t)=-\frac{1}{2}\int_{0}^{\infty }\;\;h_{t}^{2}(x)dx=-\frac{1}{2}\int_{t}^{\infty }\;\;{\left(\frac{h(x)}{\bar{H}(t)}\right)}^{2}dx$$

(5) $$=-\frac{1}{2}\int_{0}^{1}\;\;h_{t}\left({\bar{H}}_{t}^{-1}\left(u\right)\right)du.$$

The pdf of $T_{t}$ is represented by the following:

(6) $$h_{t}(x)=\frac{h\left(x+t\right)}{\bar{H}\left(t\right)},x,t>0.$$

Additionally, ${\bar{H}}_{t}^{-1}(u)=inf\left\{x;{\bar{H}}_{t}(x)\geq u\right\}$ denotes the quantile function of

(7) $$\begin{array}{c} {\bar{H}}_{t}(x)=\frac{\bar{H}\left(x+t\right)}{\bar{H}\left(t\right)},x,t>0. \end{array}$$

Extropy and its dynamic versions have attracted considerable attention in recent years, particularly in the contexts of order statistics, record values, and system reliability. Toomaj et al. [13] demonstrated that extropy effectively ranks the uniformity of a broad class of absolutely continuous distributions and highlighted several theoretical advantages of this measure over entropy. Notably, they derived a closed-form expression for the extropy of finite mixture distributions and extended the analysis to dynamic settings by introducing residual and past extropy. Building on this foundation, Qiu [14] and Qiu and Jia [15,16] investigated the extropy and residual extropy of order statistics and record values, establishing key results concerning characterization, monotonicity, and bounds. In engineering applications, Qiu et al. [17] explored the extropy of lifetimes in mixed systems under the assumption of independent and identically distributed component lifetimes. More recently, Shrahili and Kayid [18] offered new perspectives on the residual extropy of order statistics, while Saha and Kayal [19] introduced copula-based extropy measures, elucidating their connections to dependence structures.

Parallel developments have also expanded the landscape of information-theoretic tools in reliability theory. These include cumulative residual entropy, extended fractional cumulative past entropy, paired ϕ-entropy, weighted (residual) varentropy, and dynamic varentropy, among others [20,21,22,23,24]. Most recently, Kayid and Shrahili [25] analyzed consecutive *k* -out-of-*n* systems through the lens of fractional generalized cumulative residual entropy, deriving several structural properties.

Despite these advances, the residual extropy of consecutive systems—particularly consecutive *k*-out-of-*n:*G systems—remains largely unexplored. This paper fills that gap by studying the variability and uncertainty of system lifetimes using residual extropy. A key advantage of residual extropy over many alternative information measures is its computational tractability, especially for complex or composite lifetime distributions. This analytical simplicity enhances its practical utility, making it a powerful and accessible tool for quantifying uncertainty in reliability modeling.

Therefore, the remainder of this paper is organized as follows. Section 2 derives an explicit expression for the residual extropy of consecutive r-out-of-n:G systems and establishes its connection to the residual extropy of samples from a uniform distribution. Preservation properties under stochastic orderings and useful bounds are also presented. Section 3 provides several monotonicity and characterization results. Section 4 investigates the extropy of conditional consecutive r-out-of-n:G systems under the assumption that all components are functional at time t. Section 5 introduces a parametric estimator based on exponential component lifetimes for estimating the residual extropy and illustrates its performance using both simulated and real datasets. Finally, Section 6 summarizes the main findings and outlines concluding remarks, contributions and concludes the study.

Throughout this paper, we consider non-negative random variables denoted by *Z* and *Y.* These variables have absolutely cdfs denoted by $H_{Z}$ and $H_{Y}$, survival functions denoted by ${\bar{H}}_{Z}$ and ${\bar{H}}_{Y}$, and pdfs denoted by $h_{Z}$ and $h_{Y}$, respectively. The terms “increasing” and “decreasing” are used in a non-strict sense. We adopt the following notions:

- Z is less than Y in the usual stochastic order, denoted by $Z\leq_{st}Y$, if ${\bar{H}}_{Z}\left(x\right)\leq {\bar{H}}_{Y}(x)$ for all x>0;
- Z is less than Y in the hazard rate order, denoted by $Z\leq_{hr}Y$, if ${\bar{H}}_{Y}(x)/\;{\bar{H}}_{Z}\left(x\right)$ is increasing in x>0;
- Z is less than Y in the dispersive order, denoted by $Z\leq_{d}Y$, if

$$H_{Z}^{-1}\left(\beta \right)-H_{Z}^{-1}\left(\alpha \right)\leq H_{Y}^{-1}\left(\beta \right)-H_{Y}^{-1}\left(\alpha \right)\;for\;all\;0<\alpha \leq \beta <1,$$

where $H_{Z}^{-1}$ and $H_{Y}^{-1}$ are the left continuous inverses of $H_{Z}$ and $H_{Y}$, respectively. For informal definitions and properties of these notions, we refer readers to the work of Shaked and Shanthikumar [26].

## 2. Residual Extropy of Consecutive System

In the subsequent analysis, we focus on investigating the residual extropy of $T_{r|n:G}$. Uncertainty measured by the density of $\left[T_{r|n:G}-t|T_{r|n:G}>t\right]$, which shows the predictability of the residual lifetime of consecutive r-out-of-n:G systems. Let $T_{1},T_{2},\ldots ,T_{n}$ denote the lifetimes of the components of these systems, and the ordered lifetimes of the components are represented by $T_{1:n},T_{2:n},\ldots ,T_{n:n}$. Throughout this paper, we denote the lifetime of the series system as $T_{1:n}{=}_{d}T_{n|n:G}$ in contexts of interchangeability, where ${=}_{d}$ indicates equality in distribution. The residual extropy of the consecutive r-out-of-n:G system, denoted by $R\left(T_{r|n:G};t\right)$, is expressed as the extropy of $U_{r|n:G}=H\left(T_{r|n:G}\right)$. To this aim, it is known that the transformed component lifetimes $U_{i}=H\left(T_{i}\right)$ for i=1,…,n are i.i.d. random variables uniformly distributed on the interval [0,1]. So, the pdf of $U_{r|n:G}$, when 2r≥n, can be expressed as follows:

(8) $$\begin{array}{c} g_{r|n:G}(u)=r(n-r+1)(1-u{)}^{r-1}-(r+1)(n-r)(1-u{)}^{r}, \end{array}$$

where 0<u<1. The pdf (8) is derived by observing that the Jacobian of $T_{r|n:G}=H^{-1}\left(U_{r|n:G}\right)$ is given by $1/h(H^{-1}\left(u\right))$. The expression $\frac{1}{h(H^{-1}\left(u\right))}=\left|\frac{\partial H^{-1}\left(u\right)}{\partial u}\right|$ represents the Jacobian of the transformation. Consequently, we have $g_{r|n:G}(u)=h_{r|n:G}\left(H^{-1}(u)\right)/h(H^{-1}\left(u\right)),0<u<1$. On the other hand, the reliability function of $U_{r|n:G}$ can be obtained as

(9) $$\begin{array}{c} {\bar{G}}_{r|n:G}(u)=(n-r+1)(1-u{)}^{r}-(n-r)(1-u{)}^{r+1},\;for\;all\;0<u<1. \end{array}$$

In the rest of this paper, we use the notation Y∼B(t;a,b) to indicate that the random variable Y has a truncated beta distribution with the density function

(10) $$\begin{array}{c} h_{Y}(y)=\frac{1}{B\left(t;a,b\right)}y^{a-1}(1-y{)}^{b-1},0<y<t,\;for\;all\;a,b>0, \end{array}$$

where

$$B(t;a,b)=\int_{0}^{t}\;x^{a-1}(1-x{)}^{b-1},0<t<1,$$

denotes the lower incomplete beta function. The next lemma is crucial for the subsequent analyses and simplifies the computation of the residual extropy of consecutive r-out-of-n:G systems.

**Lemma** **1.** *Let* $U_{r|n:G}$*stand for the lifetime of the consecutive* r*-out-of-n:G* *system having the i.i.d. component lifetimes uniformly distributed in [0, 1]. Then, for* n/2≤r<n *, we have*

$$R\left(U_{r|n:G};t\right)=-\frac{(r(n-r+1){)}^{2r+1}B\left(\frac{\left(r+1)(n-r\right)}{r(n-r+1)}(1-t);2r-1,3\right)}{2((r+1)(n-r){)}^{2r-1}{\bar{G}}_{r|n:G}^{2}(t)},\;\mathrm{for}\;\mathrm{all}\;0<t<1$$

**Proof.**  Let us define A=r(n−r+1) and B=(r+1)(n−r). Using Equations (4), (8) and (9), is expressed as

(11) $$\begin{array}{ll} R\left(U_{r|n:G};t\right) & =-\frac{1}{2}\int_{t}^{1}\;\;{\left(\frac{g_{r|n:G}\left(u\right)}{{\bar{G}}_{r|n:G}\left(t\right)}\right)}^{2}du\\ & =-\frac{1}{2{\bar{G}}_{r|n:G}^{2}\left(t\right)}\int_{t}^{1}\;\;{\left(A(1-u{)}^{r-1}-B(1-u{)}^{r}\right)}^{2}du\\ & =-\frac{1}{2{\bar{G}}_{r|n:G}^{2}\left(t\right)}\int_{0}^{1-t}\;\;{\left(Az^{r-1}-Bz^{k}\right)}^{2}dz,\left(\;\mathrm{taking}\;z=1-u\right)\;\;\\ & =-\frac{A^{2}}{2{\bar{G}}_{r|n:G}^{2}\left(t\right)}\int_{0}^{1-t}\;\;z^{2r-2}{\left(1-\frac{B}{A}z\right)}^{2}dz\\ & =-\frac{A^{2r+1}}{2B^{2r-1}{\bar{G}}_{r|n:G}^{2}(t)}\int_{0}^{\frac{B}{A}(1-t)}\;\;w^{2r-2}(1-w{)}^{2}dw,\left(\;\mathrm{taking}\;w=\frac{B}{A}z\right)\\ & =-\frac{A^{2r+1}B\left(\frac{B}{A}\left(1-t\right);2r-1,3\right)}{2B^{2r-1}{\bar{G}}_{r|n:G}^{2}\left(t\right)}, \end{array}$$

this completes the proof. □

The lemma above covers the range n/2≤r<n. For the boundary case r=n, corresponding to the series system, the next result follows directly, and its proof is therefore omitted.

**Lemma** **2.** *If* $U_{n|n:G}$ *represents the lifetime of the series system with the i.i.d. component lifetimes uniformly distributed in [0, 1], then, for all* n≥1 *, we have*

$$R\left(U_{n|n:G};t\right)=-\frac{n^{2}B((1-t);2n-1,1)}{2(1-t{)}^{2n}},\;\mathrm{for}\;\mathrm{all}\;0<t<1$$

**Remark** **1.** *An explicit expression, as stated in Lemma 1, can be directly obtained from Equation (11) after some algebraic manipulation:*

$$\begin{array}{ll} R\left(U_{r|n:G};t\right) & =-\frac{1}{2{\bar{G}}_{r|n:G}^{2}(t)}\int_{0}^{1-t}\;\;{\left(Az^{r-1}-Bz^{k}\right)}^{2}dz\\ & =-\frac{1}{2{\bar{G}}_{r|n:G}^{2}(t)}\left(A^{2}(1-t{)}^{2r-1}+B^{2}(1-t{)}^{2r+1}-2AB(1-t{)}^{2r}\right)\\ & =-\frac{(1-t{)}^{2r-1}\left(A^{2}+B^{2}(1-t{)}^{2}-2AB(1-t)\right)}{2(1-t{)}^{2r}((n-r+1)-(n-r)(1-t){)}^{2}}\\ & =-\frac{(A-B(1-t){)}^{2}}{2(1-t)((n-r+1)-(n-r)(1-t){)}^{2}}. \end{array}$$

After simplifying, for all 2r≥n, we have the following representation

$$R\left(U_{r|n:G};t\right)=-\frac{(r(n-r+1)-(r+1)(n-r)(1-t){)}^{2}}{2(1-t)((n-r+1)-(n-r)(1-t){)}^{2}},\;\mathrm{for}\;\mathrm{all}\;0<t<1.$$

In the upcoming theorem, we express the extropy of $T_{r|n:G}$ using the previously mentioned transformations and referencing Lemma 1.

**Theorem** **1.** *Let* $T_{r|n:G}$ *stand for the lifetime of the consecutive* r *-out-of-* n:G *system having the i.i.d. component lifetimes with pdf* h *and cdf* H *, respectively. Then, for* n/2≤r<n *, we have*

(12) $$\begin{array}{c} \;\;\;\;\;\;\;\;\;\;\;\;\;\;\;\;J\left(T_{r|n:G};t\right)=R\left(U_{r|n:G};H(t)\right)E\left[h\left(H^{-1}\left(1-\frac{r(n-r+1)}{\left(r+1)(n-r\right)}Y_{r|n}\right)\right)\right], \end{array}$$

in which $Y_{r|n}∼B\left(\frac{\left(r+1)(n-r\right)}{r(n-r+1)}\bar{H}(t);2r-1,3\right)$, for all t>0.

**Proof.**  Assume that A and B are defined as in the proof of Lemma 1. In view of (1), (2) and (4) and using the change of variable u=H(x), yields the following representation:

(13) $$\;\;\;\;\;\;\;\;\;\;\;\begin{array}{lll} R\left(T_{r|n:G};t\right) & =-\frac{1}{2}\int_{t}^{\infty }\;\;{\left(\frac{h_{r|n:G}\left(x\right)}{{\bar{H}}_{r|n:G}\left(t\right)}\right)}^{2}dx & \\ & =-\frac{1}{2{\bar{H}}_{r|n:G}^{2}(t)}\int_{t}^{\infty }\;\;h^{2}(x){\left(A{\bar{H}}^{r-1}(x)-B{\bar{H}}^{r}(x)\right)}^{2}dx & \\ & \;=-\frac{1}{2{\bar{H}}_{r|n:G}^{2}\left(t\right)}\int_{H\left(t\right)}^{1}\;\;h\left(H^{-1}\left(u\right)\right){\left(A(1-u{)}^{r-1}-B(1-u{)}^{r}\right)}^{2}du & \\ & \;=-\frac{A^{2}}{2{\bar{H}}_{r|n:G}^{2}\left(t\right)}\int_{0}^{\bar{H}\left(t\right)}\;\;h\left(H^{-1}\left(1-z\right)\right)z^{2r-2}{\left(1-\frac{B}{A}z\right)}^{2}dz,\left(\;\mathrm{taking}\;z=1-u\right) & \\ & \;=-\frac{A^{2r+1}}{2B^{2r-1}{\bar{G}}_{r|n:G}^{2}\left(H\left(t\right)\right)}\int_{0}^{\frac{B}{A}\bar{H}\left(t\right)}\;\;h\left(H^{-1}\left(1-\frac{A}{B}w\right)\right)w^{2r-2}(1-w{)}^{2}dw,\left(\;\mathrm{taking}\;w=\frac{B}{A}z\right) & \\ & \;=-\frac{A^{2r+1}B\left(\frac{B}{A}\bar{H}\left(t\right);2r-1,3\right)}{2B^{2r-1}{\bar{G}}_{r|n:G}^{2}\left(H\left(t\right)\right)}\int_{0}^{\frac{B}{A}\bar{H}\left(t\right)}\;\;h\left(H^{-1}\left(1-\frac{A}{B}w\right)\right)\frac{w^{2r-2}(1-w{)}^{2}}{B\left(\frac{B}{A}\bar{H}\left(t\right);2r-1,3\right)}dw & \\ & \;=R\left(U_{r|n:G};H\left(t\right)\right)E\left[h\left(H^{-1}\left(1-\frac{r\left(n-r+1\right)}{\left(r+1\right)\left(n-r\right)}Y_{r|n}\right)\right)\right].\;\;\;\;\;\;\;\;\;\;\;\;\;\;\;\;\;\;\;\;\;\;\;\;\;\;\;\;\;\;\;\;\;\;\;\;\;\;\;\;\;\;\;\;\;\;\;\; & \end{array}$$

The final equality is an immediate consequence of Lemma 1, this completes the proof. □

In the case of a series system, the following theorem provides a formal statement of its residual extropy properties and highlights its role as a boundary case among consecutive *r*-out-of-*n*:G systems.

**Theorem** **2.** *If* $U_{n|n:G}$ *represents the lifetime of the series system having the i.i.d. component lifetimes with pdf* h *and cdf* H *, respectively, then, for all* n≥1 *, we have*

(14) $$\begin{array}{c} R\left(T_{n|n:G};t\right)=R\left(U_{n|n:G};H(t)\right)E\left[h\left(H^{-1}\left(1-Y_{n|n}\right)\right)\right], \end{array}$$

in which $Y_{n|n}∼B(\bar{H}(t);2n-1,1)$, for all t>0.

The age of the component lifetimes in a consecutive r-out-of-n:G system plays a crucial role in determining the behavior of the residual extropy lifetimes.

**Definition** **1.** *Let* T *be a nonnegative random variable with probability density function* h(x)*, survival function* $\bar{H}(x)$*, and hazard rate function defined as* $\lambda (x)=h(x)/\bar{H}(x)$*. The random variable* T *is said to exhibit an increasing failure rate (IFR) property if* λ(x) *increases with* x*, and it shows a decreasing failure rate (DFR) property if* λ(x) *decreases with* x.

The following theorem establishes the relationship between the IFR property of the parent distribution and the residual extropy of the consecutive r-out-of-n:G system lifetime. Several well-established probability distributions, including the Weibull, gamma, Pareto, exponential, and log-logistic distributions, exhibit the IFR property. Consequently, Theorem 3 is applicable to these distributions as well.

**Theorem** **3.** *If* T *is IFR, then for all* $2r\geq n,R\left(T_{r|n:G};t\right)$ *is increasing in*  t.

**Proof.**  We recall that if 2r≥n and the component lifetimes are IFR, then $T_{r|n:G}$ is IFR according to Theorem 4 of Eryilmaz and Navarro [27]. Therefore, the proof is completed using Theorem 5.3 of Toomaj et al. [13]. □

The following example illustrates the application of Theorems 1–3.

**Example** **1.** *Suppose that* $T_{r|n:G}=max\left(T_{\left[1:k\right]},T_{\left[2:k+1\right]},\ldots ,T_{\left[n-k+1:n\right]}\right)$*, such that* $T_{\left[j:m\right]}=min\left(T_{j},\ldots ,T_{m}\right)$ *for* 1≤j<m≤n *, be the lifetime of a linear consecutive* r *-out-of-* n *:G system having the i.i.d. component lifetimes follows the Rayleigh distribution with survival function given by*

(15) $$\begin{array}{c} \bar{H}(x)=e^{-\frac{x^{2}}{2}},\;x>0. \end{array}$$

The Rayleigh distribution coincides with the chi distribution with two degrees of freedom; its square is exponential. Through a straightforward calculation, it can be shown that:

$$h\left(H^{-1}(v)\right)=(1-v)\sqrt{-2log(1-v)}\;\mathrm{for}\;\mathrm{all}\;0<v<1.$$

Due to Lemma 1, we obtain

$$R\left(U_{r|n:G};H(t)\right)=-\frac{(r(n-r+1){)}^{2r+1}B\left(\frac{\left(r+1)(n-r\right)}{r(n-r+1)}e^{-\frac{t^{2}}{2}};2r-1,3\right)}{2((r+1)(n-r){)}^{2r-1}{\left({\bar{G}}_{r|n:G}\left(1-e^{-\frac{t^{2}}{2}}\right)\right)}^{2}},t>0.$$

On the other hand, one can see that

$$\begin{array}{ll} E\left[h\left(H^{-1}\left(1-\frac{r(n-r+1)}{\left(r+1)(n-r\right)}Y_{r|n}\right)\right)\right]= & \left(\frac{r(n-r+1)}{\left(r+1)(n-r\right)}\right)\\ & \times \int_{0}^{\frac{\left(r+1)(n-r\right)}{r(n-r+1)}}\;\;e^{-\frac{t^{2}}{2}}\sqrt{-2log\left(\frac{r(n-r+1)}{\left(r+1)(n-r\right)}w\right)}\\ & \times \frac{w^{2r-1}(1-w{)}^{2}}{B\left(\frac{\left(r+1)(n-r\right)}{r(n-r+1)}e^{-\frac{t^{2}}{2}};2r-1,3\right)}dw \end{array}$$

Thus, for all n/2≤r<n, Equation (12) and the previous expressions yield the following result

$$\begin{array}{ll} R\left(T_{r|n:G};t\right) & =-\frac{(r(n-r+1){)}^{2r+2}}{2((r+1)(n-r){)}^{2r}{\left((n-r+1)e^{-\frac{kt^{2}}{2}}-(n-r)e^{-\frac{(r+1)t^{2}}{2}}\right)}^{2}}\\ & \times \int_{0}^{\frac{\left(r+1)(n-r\right)}{r(n-r+1)}e^{-\frac{t^{2}}{2}}}\;\;w^{2r-1}(1-w{)}^{2}\sqrt{-2\log\left(\frac{r\left(n-r+1\right)}{\left(r+1\right)\left(n-r\right)}w\right)}dw, \end{array}$$

for all t>0. In the particular case where r=n, the residual extropy for the series system, as derived in Equation (14), is

$$R\left(T_{n|n:G};t\right)=-\frac{\sqrt{n}\Gamma \left(\frac{3}{2},nt^{2}\right)}{4e^{-nt^{2}}},$$

where Γ(a,x) is known as the upper incomplete gamma function and is defined as follows:

$$\Gamma (a,x)=\int_{x}^{\infty }\;t^{a-1}e^{-t}dt,a,x>0.$$

**Figure 1:**
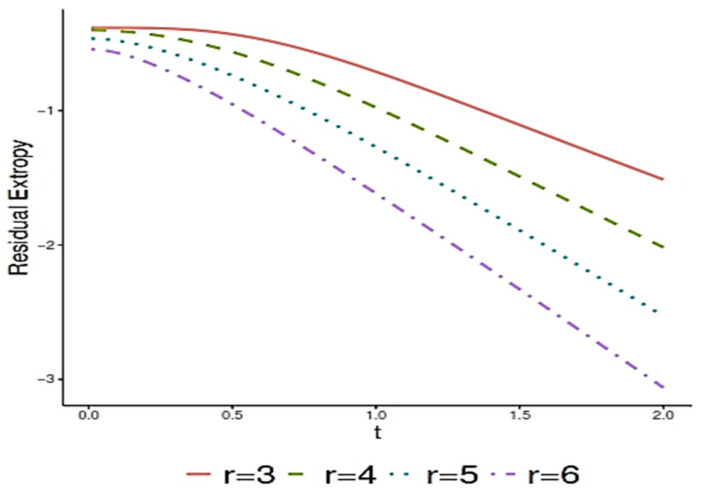
The exact values of $R\left(T_{r|6:G};t\right)$ with respect to t for the Rayleigh distribution when r=3, 4, 5, 6.

Generally, obtaining an explicit analytical expression for $R\left(T_{r|n:G};t\right)$ is a challenge. So, we employ a computational approach to investigate the behavior of $R\left(T_{r|n:G};t\right)$ for the special case n=6 and r=3, 4, 5, 6 over time t. Figure 1 summarizes the numerical analysis, illustrating the relationship between $R\left(T_{r|6:G};t\right)$ and t and values of r=3, 4, 5, 6. These trends align with Theorem 3, which shows that the residual extropy decreases with t for IFR random variables.

This decreasing trend carries important practical implications. Residual extropy quantifies the uncertainty about the remaining lifetime of a system given that it has survived up to time t. A decline in residual extropy over time indicates that the system becomes more predictable as it ages: the probability density function of the residual lifetime becomes increasingly concentrated, reducing uncertainty. In reliability terms, this aligns with the behavior of IFR systems, which are more likely to fail as they become older.

An explicit expression for the residual extropy of the series system in the Rayleigh distribution has been derived. However, a closed form for the residual extropy of consecutive r-out-of-n:G system for these distributions when n/2≤r<n. This limitation also arises for other distributions, where no closed form exists for the residual extropy of consecutive r-out-of-n:G systems. To address this challenge, we derive bounds for the residual extropy, and, in particular, establish a theorem that provides a lower bound expressed in terms of the residual extropy of the corresponding system under the uniform distribution on [0, 1] and the mode of the original distribution.

**Theorem** **4.** *Under the conditions of Theorem 1, suppose we have* M=h(m)<∞*, where* m *is the mode of the pdf* h *. Then, for all* 2r≥n *, we obtain*

$$R\left(T_{r|n:G};t\right)\geq R\left(U_{r|n:G};H\left(t\right)\right)M,for\;t>0.$$

**Proof.**  For all n/2≤r<n, it holds that

$$h\left(H^{-1}\left(1-\frac{r(n-r+1)}{\left(r+1)(n-r\right)}u\right)\right)\leq M,0<u<1,$$

thus, we have

$$E\left[h\left(H^{-1}\left(1-\frac{r(n-r+1)}{\left(r+1)(n-r\right)}Y_{r|n}\right)\right)\right]\leq M.$$

The result is readily obtained from (12) and this completes the proof. □

By applying the same reasoning and invoking Theorem 2, the lower bound for the series system can be obtained. In general, we establish a lower bound for the residual extropy of $T_{r|n:G}$ in terms of the residual extropy of a uniform r-out-of-n:G system and the mode M of the original distribution. Table 1 presents these lower bounds for several common distributions, as derived from Theorem 4.

**Figure 2:**
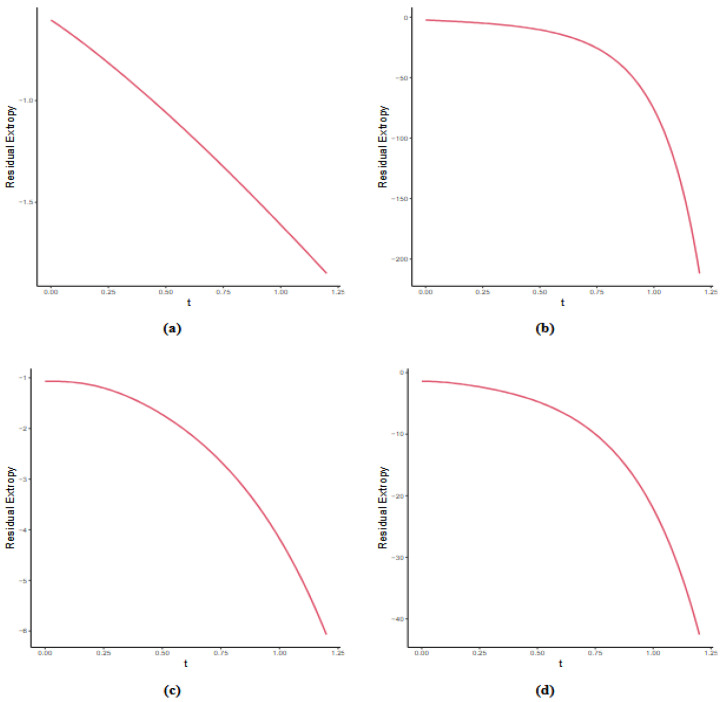
The plot of bounds for the distributions given in Table 1: (**a**) T∼HC(0, 1), (**b**) T∼HN(0, 2), (**c**) T∼GE(3, 0.5), and (**d**) T∼GG(4, 2).

In Figure 2, we present the residual extropy bounds for various parameter values of the distributions listed in Table 1.

The following theorem establishes that, for consecutive r-out-of-n:G systems with DFR components, the series system attains the minimum residual extropy. Several widely used families (e.g., Weibull, gamma, Pareto, exponential, log-logistic) admit IFR or DFR behavior under suitable parameter ranges; hence Theorem 5 applies in those cases.

**Theorem** **5.** *Let us assume that* $T_{i},i=1,2,\ldots ,n$*, denotes i.i.d. lifetimes of the components having the DFR property. Then, for all* 2r≥n

(i) it holds that $R\left(T_{1:n};t\right)\leq R\left(T_{r|n:G};t\right)$, for all t>0
(ii) it holds that $R\left(T_{1:r};t\right)\leq R\left(T_{r|n:G};t\right)$, for all t>0

**Proof.**  (i) The relation $T_{1:n}\leq_{hr}T_{r|n:G}$ is readily apparent because ${\bar{H}}_{1:n}(t)={\bar{H}}^{n}(t)$ and hence recalling (1) the function

$$\frac{{\bar{H}}_{r|n:G}(t)}{{\bar{H}}_{1:n}(t)}={\bar{H}}^{k-n}(t)(1+(n-r)H(t)),$$

is increasing in t for all 1≤r≤n. Moreover, if T demonstrates the DFR property, then $T_{1:n}$ also possesses this property. Since $T_{1:n}\leq_{hr}T_{r|n:G}$ and $T_{1:n}$ is DFR, thus utilizing Theorem 5.2. of Toomaj et al. [13], infer that $R\left(T_{1:n};t\right)\leq R\left(T_{r|n:G};t\right)$ for all t>0.

(iii) Referring to Proposition 3.2 by Navarro and Eryilmaz [28], it can be deduced that $T_{1:r}\leq_{hr}T_{r|n:G}$. Consequently, employing similar arguments as in Part (i) yields comparable outcomes, and this completes the proof. □

## 3. Monotone Properties and Characterization Results

The residual extropy of a consecutive system measures the uncertainty in its remaining lifetime, conditional on survival up to time t. Its monotonic behavior describes how this uncertainty evolves as the system ages. For consecutive r-out-of-n:G systems, these properties reveal how the predictability of failure changes over time and under different structures. A decreasing residual extropy indicates that failure becomes more predictable with age, thereby supporting more effective maintenance planning. Conversely, systems with higher residual extropy retain greater unpredictability, which may complicate scheduling but reflects more resilient behavior. Such monotonicity analysis also enables systematic comparisons across system structures and component arrangements, making it an important tool in both theoretical studies of reliability and practical extropy-based assessment. In this section, we investigate the monotonic properties of residual extropy in consecutive systems and derive related characterization results. The following theorem establishes a key monotonicity property of residual extropy in consecutive systems.

**Theorem** **6.** *For* 2r≥n*, if* $R\left(T_{1:n};t\right)$ *is decreasing in* t*, then* $R\left(T_{r|n:G};t\right)$ *is also decreasing in*  t>0.

**Proof.**  Since the hazard rate function of $T_{1:n}$ is $\lambda_{1:n}(t)=n\lambda (t)$, where λ(t) represents the hazard rate function of T, Equations (1) and (2) yield the following:

(16) $$\begin{array}{c} \frac{\lambda_{r|n:G}(t)}{\lambda_{1:n}(t)}=\Psi_{r|n:G}(\bar{H}(t)), \end{array}$$

where

$$\Psi_{r|n:G}(x)=\frac{r(n-r+1)-(r+1)(n-r)x}{n(n-r+1)-n(n-r)x},0<x<1.$$

As $\Psi_{r|n:G}(x)>0$, is strictly decreasing in x∈(0,1) for 2r≥n, thus $\Psi_{r|n:G}(\bar{H}(t))$ is strictly increasing in t. Consequently, the ratio $\lambda_{r|n:G}(t)/\lambda_{1:n}(t)$ is also strictly increasing in t. Moreover, it holds that $T_{1:n}\leq_{lr}T_{r|n:G}$. Thus, Theorem 3 of Kayid [29] directly implies the decreasing monotonicity of $R\left(T_{r|n:G};t\right)$ in t and this completes the proof. □

The following counterexample demonstrates that the result in Theorem 6 does not apply in the increasing case, meaning that even if $R\left(T_{1:n};t\right)$ increases with t, then $R\left(T_{r|n:G};t\right),t>0$, may not necessarily increase for all 2r≥n.

**Example** **2.** *Assume that* $T_{r|8:G},r=4,\;5,\;6,\;7,\;8$*, denotes the lifetime of the linear consecutive* r *-out-of-* 5:G *system. This system consists of 5 components arranged in a linear order. The system functions if and only if at least* r *consecutive components are functioning. We assume that the lifetimes of these components are i.i.d. following the Pareto type II distribution with the parameters 1 and 3 with the cdf given by*

$$H(x)=1-\frac{1}{(1+x{)}^{3}},x>0.$$

**Figure 3:**
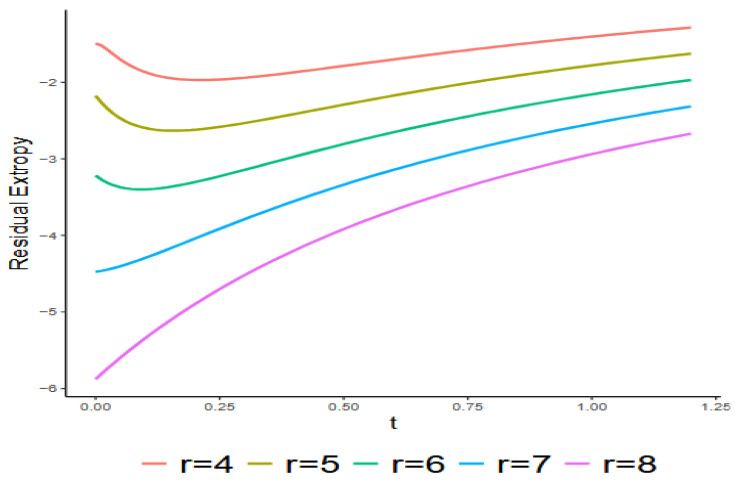
Exact values of $R\left(T_{r|8:G};t\right)$ with respect to t given in Example 2.

Figure 3 summarizes the numerical analysis showing the relationship between $R\left(T_{r|8:G};t\right)$ and t for r=4, 5, 6, 7, 8. The figure indicates that $R\left(T_{8|8:G};t\right)$ and $R\left(T_{7|8:G};t\right)$ increase as t increases, while this is not the case for $R\left(T_{r|8:G};t\right)$ when r=4, 5, 6. This suggests that an increase in $R\left(T_{1:n};t\right)$ with respect to t does not necessarily imply an increase in $R\left(T_{r|n:G};t\right)$ for all n/2≤r<n when t>0.

Recently, Qiu and Jia [16] showed that $R\left(T_{1:n};t\right)$ decreases with t if the pdf h is a decreasing function on (0,∞). However, the following counterexample demonstrates that this theorem may not always apply. Before presenting the example, we introduce a theorem that highlights the monotonicity properties of the residual extropy in series systems.

**Theorem** **7.** *If* $T_{1:n}$ *denotes the lifetime of the series system having the i.i.d. components with DFR lifetimes, then* $R\left(T_{1:n};t\right)$ *increases with* t.

**Proof.**  It is well-known that if T is DFR, then $T_{1:n}$ is also DFR, as the hazard rate function of $T_{1:n}$ is given by $\lambda_{1:n}(t)=n\lambda (t),t>0$. The proof is concluded by referencing Theorem 5.3 from Toomaj et al. [13], and this completes the proof. □

We now present a counterexample demonstrating that the result in Theorem 7 cannot be generalized to the consecutive r-out-of-n:G systems when n/2≤r<n. Furthermore, we indicate that the conclusion of Theorem 6 by Qiu and Jia [16] may not be valid.

**Example** **3.** *Let us consider a linear consecutive* r*-out-of-4:G system with lifetime* $T_{r|4:G}$ *for* r=2, 3, 4 *. The lifetimes of the components are i.i.d. with the following pdf*

$$h(x)=\frac{1}{(1+x{)}^{2}},x>0$$

**Figure 4:**
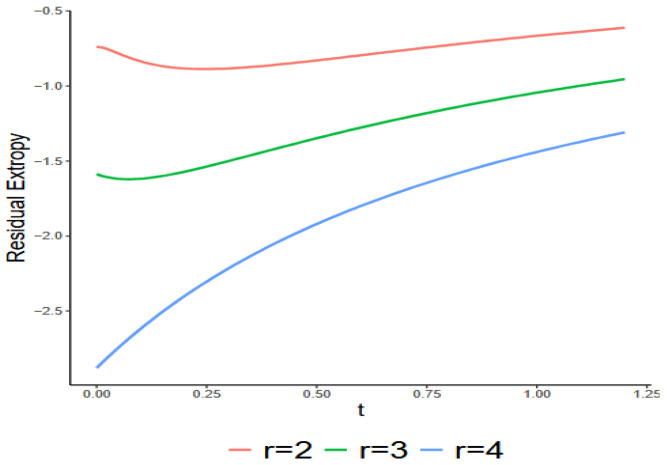
Exact values of $R\left(T_{r|4:G};t\right)$ with respect to t for r=2, 3, 4, as given in Example 3.

This follows a Log-Logistics distribution with both shape and scale parameters of unity, exhibiting the DFR property, and its pdf is decreasing in x. The numerical analysis, illustrated in Figure 4, examines the relationship between $R\left(T_{r|4:G};t\right)$ and t for r=2, 3, 4. The figure indicates that $R\left(T_{4|4:G};t\right)$ increases with t, while this behavior does not apply to $R\left(T_{2|4:G};t\right)$ and $R\left(T_{3|4:G};t\right)$. This suggests that the findings in Theorem 7 do not extend to the consecutive r-out-of-n:G systems.

Characterizing the underlying distribution is an important theme in the literature. In this context, Baratpour et al. [30] showed that the Rényi entropy of the i*th* order statistic uniquely determines the underlying distribution. Related results were obtained by Baratpour [31] for the cumulative residual entropy of the first-order statistic. Qiu [14] further demonstrated that the extropy of the i*th* order statistic provides a similar characterization, and Qiu and Jia [16] extended this to residual extropy. Motivated by these findings, we now investigate the characterization of the underlying distribution through the residual extropy of consecutive systems. Specifically, the derivative of the residual extropy can be expressed as

$$\frac{dR(Z;t)}{dt}=\frac{\lambda^{2}(t)}{2}-2\lambda (t)R(Z;t),$$

or equivalently

(17) $$\begin{array}{c} \frac{dR(Z;t)}{dt}+2\lambda (t)R(Z;t)=\frac{\lambda^{2}(t)}{2}, \end{array}$$

for all t>0, where λ(t) denotes the hazard rate function of T. To achieve our aim, we recall the problem of establishing a sufficient condition for the existence of a unique solution to the initial value problem (IVP):

(18) $$\begin{array}{c} \frac{dy}{dx}=h(x,y),y\left(x_{0}\right)=y_{0}, \end{array}$$

where h is a function of two variables defined in a region $D\subseteq R^{2}$, and ($x_{0},y_{0}$) is a point in D. Here, y is the unknown function. By the solution of (18), we find a function ϕ(⋅) which satisfies the following conditions:

(i) ϕ(⋅) is differentiable on I⊆R,
(ii) the growth of ϕ(⋅) lies in D,
(iii) $\phi \left(x_{0}\right)=y_{0}$ and (iv) $\phi^{’}(x)=h(x,\phi (x))$, for all x∈I.

The next theorem and lemma, originally presented by Gupta and Kirmani [32], will be utilized in deriving our characterization results.

**Theorem** **8.** *Let* h *be a continuous function defined in a domain* $D\subseteq R^{2}$ *, and let* h *satisfy Lipschitz condition (with respect to* y *) in* D *, that is* $\left|f\left(x,y_{1}\right)-f\left(x,y_{2}\right)\right|\leq r\left|y_{1}-y_{2}\right|,k>0$ *, for every point (* $x,y_{1}$ *) and (* $x,y_{2}$ *) in* D *. Then, the function* y=ϕ(x) *satisfying the IVP* $y^{\prime }=h(x,y)$ *and* $y\left(x_{0}\right)=y_{0},x\in I$ *, is unique.*

**Lemma** **3.** *Suppose that the function* h *is continuous in a convex region*  $D\cap R^{2},\frac{\partial f}{\partial y}$ *exists and is continuous in* D*. Then* h *satisfies the Lipschitz condition in*  D.

We conclude this section with a characterization of the underlying distribution based on the residual extropy of consecutive r-out-of- n:G systems.

**Theorem** **9.** *Let* $R\left(T_{r|n:G}^{Z};t\right)$ *and* $R\left(T_{r|n:G}^{Y};t\right)$ *be the residual extropy of the consecutive* *r-out-of-n:*G *system having the i.i.d. component lifetimes* $Z_{i}$ *and* $Y_{i}$ *with pdfs* $h_{Z}$ *and* $h_{Y}$*, and cdfs* $H_{Z}$ *and* $H_{Y}$*, respectively. Then* $Z\overset{d}{=}Y$ *if and only if for all* $2r\geq n,R\left(T_{r|n:G}^{Z};t\right)=R\left(T_{r|n:G}^{Y};t\right)$ *for* t≥0.

**Proof.**  We just prove the sufficiency part where the necessity is trivial. From (17), we have

$$\frac{dR\left(T_{r|n:G}^{Z};t\right)}{dt}+2\lambda_{Z,k|n:G}(t)R\left(T_{r|n:G}^{Z};t\right)=\frac{\lambda_{Z,k|n:G}^{2}(t)}{2},t>0$$

Taking the derivative of the above equation concerning t, we have

$$\frac{d\lambda_{Z,k|n:G}(t)}{dt}=\frac{\frac{d^{2}R\left(T_{r|n:G}^{Z};t\right)}{dt^{2}}+2\lambda_{Z,k|n:G}(t)\frac{dR\left(T_{r|n:G}^{Z};t\right)}{dt}}{\lambda_{Z,k|n:G}(t)-2R\left(T_{r|n:G}^{Z};t\right)},t>0$$

Assume that $R\left(T_{r|n:G}^{Z};t\right)=R\left(T_{r|n:G}^{Y};t\right)=w(t)$ for all t≥0, and 2r≥n. Then, for all t≥0, we get

$$\frac{d\lambda_{Z,k|n:G}(t)}{dt}=\Psi \left(t,\lambda_{Z,k|n:G}(t)\right),\frac{d\lambda_{Y,k|n:G}(t)}{dt}=\Psi \left(t,\lambda_{Y,k|n:G}(t)\right),$$

where

$$\Psi (t,y)=\frac{w^{\prime \prime }(t)+2zw^{\prime }(t)}{z-2w(t)},t>0$$

It follows from Theorem 8 and Lemma 3 that for all t≥0, which implies

$${\bar{H}}_{r|n:G}^{Z}(t)={\bar{H}}_{r|n:G}^{Y}(t),\;\mathrm{for}\;\mathrm{all}\;t\geq 0$$

In view of $H_{Z}(t)={\bar{G}}_{r|n:G}^{-1}\left({\bar{H}}_{r|n:G}^{Z}(t)\right)$ and $H_{Y}\left(t\right)={\bar{G}}_{r|n:G}^{-1}\left({\bar{H}}_{r|n:G}^{Y}\left(t\right)\right),$ for all t≥0, where ${\bar{G}}_{r|n:G}(\cdot )$ is defined in (9), we have $H_{Z}(t)=H_{Y}(t)$ for all t≥0, this completes the proof. □

## 4. Conditional Residual Extropy of Consecutive Systems

In this section, we aim to evaluate the residual lifetime $T_{r|n:G}-t,t\geq 0$ under the condition that all components of the system are alive at time t>0. Then, it can be seen that the survival function of $T_{n,G}^{r}(t)=\left[T_{r|n:G}-t|T_{1:n}>t\right]$ can be written as follows (see Salehi et al. [33]):

(19) $$\begin{array}{ll} {\bar{H}}_{r|n:G}(x;t) & =P\left(T_{r|n:G}-t>x|T_{1:n}>t\right)\\ & =\left(n-r+1\right){\bar{H}}_{t}^{r}\left(x\right)-\left(n-r\right){\bar{H}}_{t}^{r+1}\left(x\right),x,t>0, \end{array}$$

where ${\bar{H}}_{t}(x)$ is defined in (7). It is worth mentioning that Salehi et al. [33] studied the stochastic and aging properties of the residual lifetime of consecutive r-out-of- n systems. This was under the condition that at time t,n−r+1, where r is less than or equal to n, components of the system are in working condition. Additionally, they proposed the mean residual lifetime for such systems and derived various properties. Assuming that H is absolutely continuous with the pdf h, the pdf of $T_{n,G}^{r}(t)$ is given by

$$h_{r|n:G}\left(x;t\right)=-\frac{d{\bar{H}}_{r|n:G}\left(x;t\right)}{dx},\;for\;all\;t>0.$$

Given that

$$h_{t}\left(x\right)=-\frac{d{\bar{H}}_{t}\left(x\right)}{dx},\;for\;all\;t>0,$$

we have

(20) $$\begin{array}{c} h_{r|n:G}(x;t)=h_{t}(x)\left[r\left(n-r+1\right){\bar{H}}_{t}^{r-1}\left(x\right)-\left(r+1\right)\left(n-r\right){\bar{H}}_{t}^{r}\left(x\right)\right],x,t>0, \end{array}$$

where $h_{t}(x)$ is defined in (6). In the next, we focus on the study of the extropy of the random variable $T_{n,G}^{r}(t)$ which measures the amount of uncertainty present in the density of $\left[T_{r|n:G}-\right.\left.t|T_{1:n}>t\right]$ with regard to the predictability of the system’s residual lifetime in terms of the extropy. The probability integral transformation $U_{r|n:G}={\bar{H}}_{t}\left(T_{n,G}^{r}(t)\right)$ plays a crucial role in our investigation. The pdf of $U_{r|n:G}$ is provided in (8). In the upcoming theorem, we express the extropy of $T_{n,G}^{r}(t)$ using the aforementioned transforms.

**Theorem** **10.** *Let* $Z_{i}$ *denote i.i.d. random variables representing the lifetimes of the components of the consecutive r-out-of-n:* G *system having the common* pdfh *and cdf* H *. Then, for all* 2r≥n *, the extropy of* $T_{n,G}^{r}(t)$ *can be expressed as follows:*

(21) $$\begin{array}{c} R\left(T_{n,G}^{r}(t)\right)=-\frac{1}{2}\int_{0}^{1}\;\;g_{r|n:G}^{2}(u)h_{t}\left({\bar{H}}_{t}^{-1}\left(u\right)\right)du,\;for\;all\;t>0. \end{array}$$

**Proof.**  Setting $u={\bar{H}}_{t}(x)$ to (4) and (20) yields

$$\begin{array}{ll} R\left(T_{n,G}^{r}(t)\right) & =-\frac{1}{2}\int_{0}^{\infty }\;\;{\left(h_{T_{n,G}^{r}(t)}(x)\right)}^{2}dx\\ & =-\frac{1}{2}\int_{0}^{\infty }\;\;h_{t}^{2}(x){\left[r(n-r+1){\bar{H}}_{t}^{r-1}(x)-(r+1)(n-r){\bar{H}}_{t}^{r}(x)\right]}^{2}dx\\ & =-\frac{1}{2}\int_{0}^{1}\;\;{\left(r(n-r+1)u^{r-1}-(r+1)(n-r)u^{r}\right)}^{2}h_{t}\left({\bar{H}}_{t}^{-1}(u)\right)dx\\ & =-\frac{1}{2}\int_{0}^{1}\;\;g_{r|n:G}^{2}\left(u\right)h_{t}\left({\bar{H}}_{t}^{-1}\left(u\right)\right)du. \end{array}$$

In Equation (21), $g_{r|n:G}(u)$ represents the pdf of $U_{r|n:G}$ as given in (8), this completes the proof. □

The survival function ${\bar{H}}_{r|n:G}(x;t)$ provides the probability that the system survives beyond x, given that all components are functioning at time t. However, the conditional residual extropy offers a more comprehensive assessment of the uncertainty associated with the remaining lifetime. Traditional measures, such as the mean residual life, may not adequately capture the randomness inherent in the failure process. Extropy, as an information measure, quantifies the degree of disorder or uncertainty in the conditional residual lifetime distribution. This perspective is particularly important for consecutive systems, whose complex structure can generate non-intuitive aging patterns. The proposed measure therefore serves as a useful tool for characterizing and comparing dynamic uncertainty across different operational conditions and system configurations, providing insights beyond those offered by classical reliability metrics.

In the next theorem, we investigate how the residual extropy of the conditional consecutive system lifetime varies with the components’ aging properties.

**Theorem** **11.** *For all* 2r≥n*, if* T *is IFR (DFR), then*  $R\left(T_{n,G}^{r}(t)\right)$ *is decreasing (increasing) in* t.

**Proof.**  The proof for the IFR case extends in the same manner to the DFR case. Accordingly, $h_{t}\left({\bar{H}}_{t}^{-1}(u)\right)=u\lambda_{t}\left({\bar{H}}_{t}^{-1}(u)\right),0<u<1$, where $\lambda_{t}(x)=h_{t}(x)/{\bar{H}}_{t}(x),x,t>0$, is the hazard rate function of $T_{t}$. On the other hand, one can conclude that ${\bar{H}}_{t}^{-1}(u)={\bar{H}}^{-1}(u\bar{H}(t))-t$, for all 0<u<1, and hence we have

(22) $$\begin{array}{c} \lambda_{t}\left({\bar{H}}_{t}^{-1}(u)\right)=\lambda \left({\bar{H}}_{t}^{-1}(u)+t\right)=\lambda \left({\bar{H}}^{-1}(u\bar{H}(t))\right),0<u<1. \end{array}$$

If $t_{1}\leq t_{2}$, then ${\bar{H}}^{-1}\left(u\bar{H}\left(t_{1}\right)\right)\leq {\bar{H}}^{-1}\left(u\bar{H}\left(t_{2}\right)\right)$. This implies that Equation (21) represent as

$$\begin{array}{ll} \int_{0}^{1}\;\;{\left(g_{r|n:G}(u)\right)}^{2}u\lambda_{t_{1}}\left({\bar{H}}_{t_{1}}^{-1}(u)\right)du & =\int_{0}^{1}\;\;{\left(g_{r|n:G}(u)\right)}^{2}u\lambda \left({\bar{H}}^{-1}\left(u\bar{H}\left(t_{1}\right)\right)\right)du\\ & \leq \int_{0}^{1}\;\;{\left(g_{r|n:G}(u)\right)}^{2}u\lambda \left({\bar{H}}^{-1}\left(u\bar{H}\left(t_{2}\right)\right)\right)du\\ & =\int_{0}^{1}\;\;{\left(g_{r|n:G}(u)\right)}^{2}u\lambda_{t_{2}}\left({\bar{H}}_{t_{2}}^{-1}(u)\right)du, \end{array}$$

for all $t_{1}\leq t_{2}$. This implies that $R\left(Z_{n,t_{1}}\right)\geq R\left(Z_{n,t_{2}}\right)$, this completes the proof. □

The following example illustrates the theoretical results established in Theorems 10 and 11.

**Example** **4.** *Let us assume a linear consecutive 3-out-of-*6:G *system with lifetime*

$$T_{3|6:G}=max\left(min\left(Z_{1},Z_{2},Z_{3}\right),min\left(Z_{2},Z_{3},Z_{4}\right),min\left(Z_{3},Z_{4},Z_{5}\right),min\left(Z_{4},Z_{5},Z_{6}\right)\right).$$

**Figure 5:**
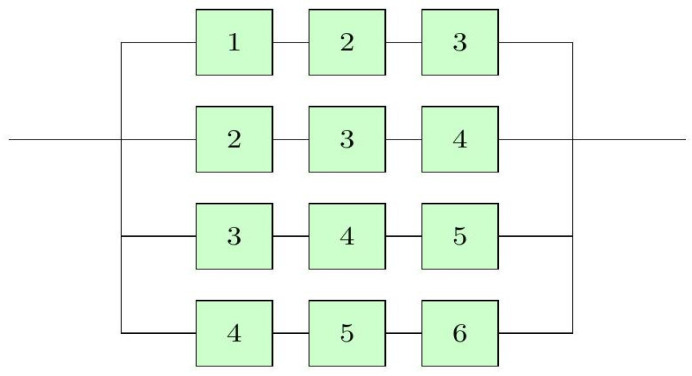
Linear consecutive 3-out-of-6:G system.

Figure 5 presents the configuration of this system, in which system reliability depends on the successful operation of at least three contiguous components.

The exact value of $R\left(T_{6,G}^{3}(t)\right)$ can be computed using Equation (21) when the component lifetime distributions are given. To this aim, let us suppose the following lifetime distributions.

(i) Assume that the component lifetimes are i.i.d. Pareto Type II with the survival function

$$\bar{H}(t)=(1+t{)}^{-b},b,t>0$$

Using this, it follows that

$$R\left(T_{6,G}^{3}(t)\right)=-\frac{1}{2}\left(\frac{b}{1+t}\right)\int_{0}^{1}\;u^{\frac{b+1}{b}}g_{r|n:G}^{2}(u)du,t>0.$$

**Figure 6:**
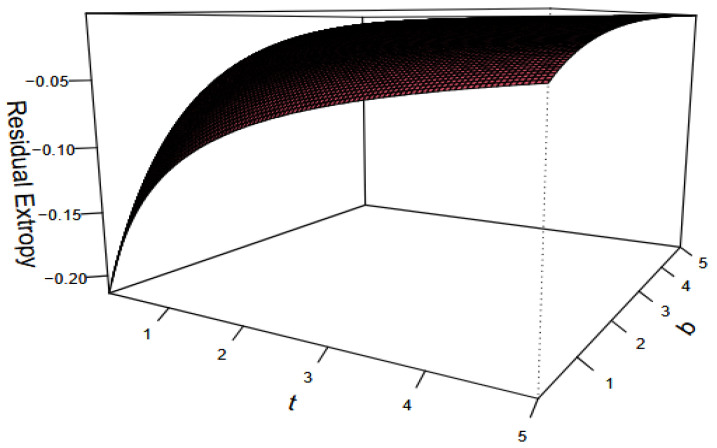
Exact values of $R\left(T_{6,G}^{3}(t)\right)$ for the Pareto Type II distribution with b,t∈(0,5).

It follows from Figure 6 that $T_{6,G}^{3}(t)$ is a monotonically increasing function with respect to both time t and the parameter b. This result suggests that as time t increases, the uncertainty about the remaining lifetime of the system, represented by $T_{6,G}^{3}(t)$ increases. Additionally, we observe that the distribution of the system component lifetimes exhibits the DFR property.

(ii) Assume T follows a Weibull distribution with shape parameter a and the given survival function

$$\bar{H}(t)=e^{-t^{a}},t>0,a>0.$$

Upon algebraic simplification, we arrive at the following expression

$$R\left(T_{6,G}^{3}(t)\right)=-\frac{a}{2}\int_{0}^{1}\;{\left(t^{a}-logu\right)}^{\left(1-\frac{1}{a}\right)}ug_{r|n:G}^{2}(u)du,t>0.$$

**Figure 7:**
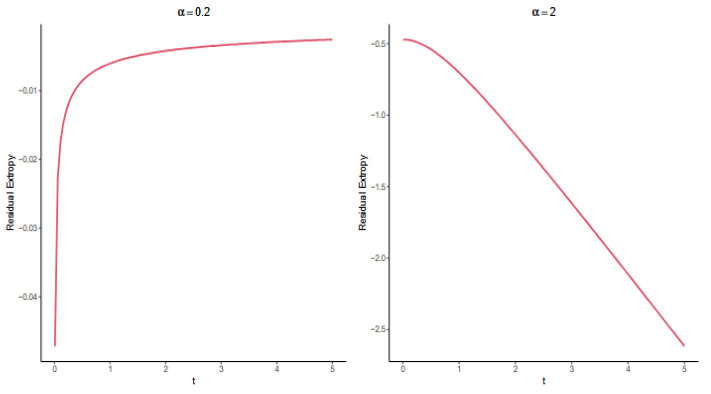
Exact values of $R\left(T_{6,G}^{3}(t)\right)$ for the Weibull distribution with α=0.2 and α=2.

An explicit expression for the aforementioned relation is intractable. Consequently, a numerical analysis is employed. Figure 7 above shows $R\left(T_{6,G}^{3}(t)\right)$ with respect to time t for a=0.2,a=2. In accordance with Theorem 11, the function $R\left(T_{6,G}^{3}(t)\right)$ exhibits an increasing trend for DFR cases 0<a<1 and a decreasing trend for IFR cases a>1. These findings are graphically represented in Figure 7.

The next theorem shows the relationship between $R\left(T_{n,G}^{r}(t)\right)$ and $R\left(T_{r|n:G}\right)$ under specific aging conditions.

**Theorem** **12.** *For all* 2r≥n*, if* T *is IFR (DFR), then*  $R\left(T_{n,G}^{r}(t)\right)\leq (\geq )R\left(T_{r|n:G}\right)$ *for all* t>0.

**Proof.**  According to Theorem 11, if T exhibits an IFR (DFR) property, then $R\left(T_{n,G}^{r}(t)\right)$ is a monotonically decreasing (increasing) function of time t. This implies that $R\left(T_{n,G}^{r}(t)\right)$ is consistently less than or equal to (greater than or equal to) $R\left(T_{r|n:G}\right)$ for all non-negative values of t, this completes the proof. □

The next theorem establishes a comparison of the stochastic ordering properties of conditional residual extropy lifetimes in consecutive r-out-of-n:G systems, under the condition that all components operate beyond time t >0.

**Theorem** **13.** *Let* $T_{n,G}^{r,Z}(t)=\left[T_{r|n:G}^{Z}-t|T_{1:n}>t\right]$ *and* $T_{n,G}^{r,Y}(t)=\left[T_{r|n:G}^{Y}-t|Y_{1:n}>t\right]$ *denote two residual lifetimes of consecutive* r*-out-of-*n *systems having*  n *i.i.d component lifetimes*  $Z_{i}$ *and* $Y_{i}$ *from cdfs*  $H_{Z}$  *and*  $H_{Y}$*, respectively. If*  $Z\leq_{d}Y$  *and*  Z  *or* Y  *is IFR, then*  $R\left(T_{n,G}^{r,Z}(t)\right)\leq R\left(T_{n,G}^{r,Y}(t)\right)$  *for all*  t>0.

**Proof.**  Recalling Equation (21), it is sufficient to show that $T_{t}$ is stochastically dominated by $Y_{t}$ in the dispersive order. Leveraging the assumption that $Z\leq_{d}Y$ and one of these random variables has IFR property, it follows by directly applying the proof technique outlined in Theorem 5 of [34] to conclude that $T_{t}\leq_{d}Y_{t},$ which completes the proof. □

**Example** **5.** *Assume two residual lifetimes* $T_{n,G}^{r,Z}(t)$ *and* $T_{n,G}^{r,Y}(t)$ *based on* n *i.i.d. component lifetimes* $T_{i}$ *and* $Y_{i}$ *for* i=1,2,…,n *, with the survival functions* ${\bar{H}}_{Z}$ *and* ${\bar{H}}_{Y}$ *, respectively. Let* Z *have a linear failure rate function with the survival function given by*

$${\bar{H}}_{Z}(x)=e^{-b_{1}x-b_{2}x^{2}/2},x,b_{1},b_{2}>0.$$

Further assume that Y is an exponential distribution with the survival function ${\bar{H}}_{Y}(x)=e^{-b_{1}x},x,b_{1}>0$. It can be seen that the hazard rate function of Z is $\lambda_{Z}(x)=b_{1}+b_{2}x$ and the hazard rate function of Y is $\lambda_{Y}(x)=b_{1}$. Since $\lambda_{Z}(x)\geq \lambda_{Y}(x),x>0$, it follows that $Z\leq_{hr}Y$. So, one can conclude that $Z\leq_{d}Y$ based on the DFR property of Z. Furthermore, both Z and Y have IFR property. Consequently, Theorem 13 implies that $R\left(T_{n,G}^{r,Z}(t)\right)\leq R\left(T_{n,G}^{r,Y}(t)\right)$ for all t>0.

Finally, we demonstrate that the extropy of the lifetime of consecutive r-out-of- n systems, under the assumption that all components are operational at time t>0, uniquely determines the distribution function. To this end, our analysis focuses on a specific system configuration: the linear consecutive (n−i)-out-of-n:G system, subject to the condition n≥2i, where i ranges from 0 to n/2. For this purpose, we first recall the Muntz–Szász theorem, as presented in Higgins [35], which will be employed to establish the main results that follow.

**Lemma** **4.** *For an integrable function* ψ(x) *on the finite interval* (a,b) *if* $\int_{a}^{b}\;x^{n_{j}}\psi (x)dx=0,j\geq 1$*, then* ψ(x)=0 *for almost all* x∈(a,b)*, where* $\left\{n_{j},j\geq 1\right\}$ *is a strictly increasing sequence of positive integers satisfying*  $\sum_{j=1}^{\infty }\;\frac{1}{n_{j}}=\infty$.

It is worth pointing out that Lemma 4 is a well-established concept in functional analysis, stating that the sets $\left\{x^{n_{1}},x^{n_{2}},\ldots ;1\leq n_{1}<n_{2}<\cdots \right\}$ constitutes a complete sequence. Notably, Hwang and Lin [36] expanded the scope of the Müntz–Szász theorem for the functions $\left\{\phi^{n_{j}}(x),n_{j}\geq 1\right\}$, where ϕ(x) is both absolutely continuous and monotonic over the interval (a,b). This lemma leads us to characterize uniquely the parent distribution using $T_{n,G}^{r}(t)$.

**Theorem** **14.** *Let* $T_{n,G}^{n-i,Z}(t)=\left[T_{n-i|n:G}^{Z}-t|T_{1:n}>t\right]$ *and* $T_{n,G}^{n-i,Y}(t)=\left[T_{n-i|n:G}^{Y}-t|Y_{1:n}>t\right]$ *denote two residual lifetimes of consecutive (* n−i *)-out-of-* n:G *systems having* n *i.i.d component lifetimes* $Z_{j}$ *and* $Y_{j}$ *from cdfs* $H_{Z}$ *and* $H_{Y}$ *, respectively. Then* $H_{Z}$ *and* $H_{Y}$ *belong to the same family of distributions if and only if for fixed* i

$$R\left(T_{n,G}^{n-i,Z}(t)\right)=R\left(T_{n,G}^{n-i,Y}(t)\right),\;\mathrm{for}\;\mathrm{all}\;n\geq 2i\;\mathrm{and}\;i=0,1,\ldots ,n/2.$$

**Proof.**  We just prove the sufficiency part where the necessity is trivial. Relation (21) for the lifetime Z can be rewritten as follows:

(23) $$\begin{array}{c} R\left(T_{n,G}^{n-i,Z}(t)\right)=-\frac{1}{2}\int_{0}^{1}\;\;g_{n-i|n:G}^{2}\left(u\right)h_{t}\left({\bar{H}}_{t}^{-1}\left(u\right)\right)du, \end{array}$$

for all t>0 and 1≤i≤n. The same argument also holds for Y. Given the assumption that $R\left(T_{n,G}^{n-i,Z}(t)\right)=R\left(T_{n,G}^{n-i,Y}(t)\right)$, Equation (23) yields

$$\int_{0}^{1}\;g_{n-i|n:G}^{2}\left(u\right)\left(h_{Z,t}\left({\bar{H}}_{Z,t}^{-1}\left(u\right)\right)-h_{Y,t}\left({\bar{H}}_{Y,t}^{-1}\left(u\right)\right)\right)du=0,for\;all\;t>0.$$

Thus, it holds that

(24) $$\begin{array}{c} \int_{0}^{1}\;\;(1-u{)}^{n-2i}\phi_{i,n}(u)\left(h_{Z,t}\left({\bar{H}}_{Z,t}^{-1}\left(u\right)\right)-h_{Y,t}\left({\bar{H}}_{Y,t}^{-1}\left(u\right)\right)\right)du=0, \end{array}$$

where

$$\phi_{i,n}(u)=(1-u{)}^{n}{\left[\left(n-i)(i+1)(1-u{)}^{-1}-i(n-i+1\right)\right]}^{2},0<u<1.$$

By taking z=1−u, Equation (24) can be rewritten as follows:

(25) $$\begin{array}{c} \;\;\;\;\;\;\;\;\;\;\int_{0}^{1}\;\;z^{n-2i}\phi_{i,n}(1-z)\left(h_{Z,t}\left({\bar{H}}_{Z,t}^{-1}\left(1-z\right)\right)-h_{Y,t}\left({\bar{H}}_{Y,t}^{-1}\left(1-z\right)\right)\right)du=0. \end{array}$$

By applying Lemma 4 with the function

$$\psi \left(x\right)=\phi_{i,n}\left(1-z\right)\left(h_{Z,t}\left({\bar{H}}_{Z,t}^{-1}\left(1-z\right)\right)-h_{Y,t}\left({\bar{H}}_{Y,t}^{-1}\left(1-z\right)\right)\right),$$

and considering the complete sequence $\left\{z^{n-2i},n\geq 1\right\}$, one can conclude that

$$h_{Z,t}\left({\bar{H}}_{Z,t}^{-1}(1-z)\right)=h_{Y,t}\left({\bar{H}}_{Y,t}^{-1}(1-z)\right),\;a.e.\;z\in (0,1),$$

or equivalently $h_{Z,t}\left({\bar{H}}_{Z,t}^{-1}(x)\right)=h_{Y,t}\left({\bar{H}}_{Y,t}^{-1}(x)\right)$ for all x∈(0,1). Since $d{\bar{H}}_{t}^{-1}(u)=-1/h_{t}\left({\bar{H}}_{t}^{-1}(u)\right)du$, it follows that ${\bar{H}}_{Z,t}^{-1}(u)={\bar{H}}_{Y,t}^{-1}(u)$ for all 0<u<1. Letting $z={\bar{H}}_{Z,t}^{-1}(u)$ implies ${\bar{H}}_{Z,t}(z)=u$. Since ${\bar{H}}_{Z,t}(z)=\frac{{\bar{H}}_{X}(z+t)}{{\bar{H}}_{X}(t)}$, (the same argument holds for Y) we find $\frac{{\bar{H}}_{Z}(z+t)}{{\bar{H}}_{Z}(t)}=\frac{{\bar{H}}_{Y}(z+t)}{{\bar{H}}_{Y}(t)}$. Since this is true for all x,t≥0, setting t=0, we conclude ${\bar{H}}_{Z}(z)={\bar{H}}_{Y}(z),z>0$, demonstrating that Z and Y have the same distribution functions, which completes the proof. □

## 5. Numerical Analysis

This section presents a simulation study of residual extropy in consecutive *r*-out-of-*n*:G systems, analyzed via the maximum likelihood estimator (MLE). To this aim, we consider random samples $T_{1},T_{2},\ldots ,T_{N}$ from exponential, Rayleigh, and Lomax distributions, and obtain the MLE for the parameter λ. The results are shown in Table 2.

By applying Equations (1), (2), and (4), the corresponding formulation for the residual extropy of these systems is obtained as follows:

$$\begin{array}{ll} R\left(T_{r|n:G};t\right) & \;=-\frac{1}{2}\int_{t}^{\infty }\;\;{\left(\frac{h_{r|n:G}(x;\lambda )}{{\bar{H}}_{r|n:G}(t;\lambda )}\right)}^{2}dx\\ & \;=-\frac{1}{2}\int_{t}^{\infty }\;\;{\left(\frac{h\left(x;\lambda \right)g_{r|n:G}\left(H\left(x;\lambda \right)\right)}{{\bar{G}}_{r|n:G}\left(H\left(t;\lambda \right)\right)}\right)}^{2}dx, \end{array}$$

for all t>0. To evaluate the efficacy of our proposed estimator for $R\left(T_{r|n:G};t\right)$ when applied to simulated data for the three distributions, we calculate both its average bias and root mean squared error (RMSE). By exploiting the invariance property of the MLE, an estimate of $R\left(T_{r|n:G};t\right)$ under such three distributions can be obtained directly, yielding the following expression:

(26) $$\;\begin{array}{rrr} \hat{R}\left(T_{r|n:G};t\right) & =-\frac{1}{2}\int_{t}^{\infty }\;\;{\left(\frac{h\left(x;\hat{\lambda }\right)g_{r|n:G}\left(H\left(x;\hat{\lambda }\right)\right)}{{\bar{G}}_{r|n:G}\left(H\left(x;\hat{\lambda }\right)\right)}\right)}^{2}dx,\;\;\;\;\;\;\;\;\;\;\;\;\;\;\;\;\;\;\;\;\;\;\;\; & \end{array}$$

for all t>0, where $h\left(x;\hat{\lambda }\right)$ and $H\left(x;\hat{\lambda }\right)$ denote the pdf and cdf of any three distributions as determined by the MLE of the parameter. To assess the performance of the estimator, we computed the bias and root mean square error (RMSE) for different sample sizes (N = 20, 30, 40, 50) and various configurations of the system parameters n,r, and t. Each case was evaluated using 5000 independent replications, and the results are summarized in Table A1, Table A2, Table A3, Table A4, Table A5, Table A6, Table A7, Table A8 and Table A9, which are provided in Appendix A.

The simulation results presented in this study provide valuable insights into how the residual extropy estimation accuracy, measured by bias and RMSE, is influenced by three key factors: total sample size *N*, the number of component lifetimes of the system *n*, and the number of working components *r*. Our findings consistently demonstrate that the estimators for the three distributions are asymptotically well-behaved: both bias and RMSE decline monotonically as the total sample size *N* increases across all configurations of *n* and *r*. This pattern supports the theoretical property of consistency, indicating that the estimator converges to the true parameter value as more data become available. A second salient pattern is the strong influence of the number of working components *r*. When both N and n are fixed, then as *r* increases, from 0.1 to 2.0, the bias and RMSE of estimators increase for the exponential and Weibull distributions; however, for the Lomax distribution, it is the converse. This result underscores the number of working components *r* in determining estimator performance, particularly in small-sample settings. Nonetheless, the present findings provide strong empirical support for the reliability of the proposed estimator under realistic finite-sample conditions and clarify the interplay between sample size and number of working components in determining estimation accuracy.

It is important to note that the maximum likelihood estimator (MLE) derived in this study relies on exponential lifetime assumptions, which ensure analytical tractability and closed-form expressions. However, in practice, deviations from exponentiality may affect both efficiency and small-sample performance. To address this, robustness checks can be carried out by applying the estimator under alternative lifetime models such as Weibull, gamma, or Lomax, and comparing the resulting bias and variance. In addition, bootstrap resampling offers a distribution-free approach to assess variability and construct confidence intervals, while Bayesian methods provide a flexible alternative that incorporates prior information and yields full posterior distributions for residual extropy. These considerations suggest that, although the exponential case serves as a useful benchmark, complementary approaches are available to extend the robustness and applicability of the proposed framework.

We now illustrate the performance of the estimator on real data, assuming an exponential distribution.

**Example** **6.** *The air conditioning system in commercial aircraft, such as the Boeing 720, plays a vital role in ensuring passenger comfort and cooling avionic components. Failures in this system can lead to operational disruptions and safety issues, making accurate reliability modeling crucial. The exponential distribution is often employed in these scenarios, based on the assumption of a constant hazard rate, which is typically applicable during the mid-life phase of complex systems. In this context, we examine the performance of the air conditioning equipment in the Boeing 720 using a dataset comprising 35 observed failure times, which supports the applicability of exponential modeling. Shanker et al. [37] investigated the suitability of the exponential distribution for analyzing this type of reliability data. The individual time intervals are as follows:*

**Dataset:** 11, 35, 49, 170, 329, 381, 708, 958, 1062, 1167, 1594, 1925, 1990, 2223, 2327, 2400, 2451, 2471, 2551, 2565, 2568, 2694, 2702, 2761, 2831, 3034, 3059, 3112, 3214, 3478, 3504, 4329, 6367, 6976, 7846, 13403.
  Jose and Sathar [38] analyzed this dataset and concluded that the exponential distribution provides an adequate fit, estimating the failure rate as $\hat{\lambda }=0.00825$. In the present study, we evaluate the estimation of the residual extropy of the consecutive *r*-out-of-*n*:G system $R\left(T_{r|6:G};t\right)$ under the exponential model with the estimated $\hat{\lambda }=0.00825$. Table 3 reports both the theoretical values $R\left(T_{r|6:G};t\right)$ and their empirical counterparts $\hat{R}\left(T_{r|6:G};t\right)$ for r=3,4,5,6 computed directly from the observed failure data. Several important observations emerge from Table 3: Across all values of t∈{0.1,0.5,0.9,1.2,2.0}, the empirical estimates $\hat{R}\left(T_{r|6:G};t\right)$ closely track the theoretical predictions $R\left(T_{r|6:G};t\right)$, supporting the adequacy of the exponential model for this dataset. As the required number of functioning components r increases (from 3 to 6), the $R\left(T_{r|6:G};t\right)$ decreases monotonically for any fixed *t*. This is intuitive: demanding more components to survive simultaneously reduces system uncertainty. The empirical estimates reflect this trend consistently. The presented results show a correlation between both the theoretical and empirical estimators.

## 6. Conclusions

This study introduced an information-theoretic reliability inference framework for consecutive *r*-out-of-*n*:G systems based on residual extropy. Explicit representations were derived in tractable cases such as the uniform distribution, while for complex lifetime models we established bounds that serve as practical tools when closed-form solutions are unavailable. These bounds are particularly valuable in applications involving mixtures or heavy-tailed distributions, where they support screening, reliability planning, and maintenance decision-making without requiring full analytic evaluation. Additional contributions include results on preservation properties under stochastic orders and aging assumptions, together with monotonicity and characterization findings. A conditional framework, in which all components are assumed operational at a given time, was also examined, offering further insights into the behavior of multicomponent systems. Collectively, these results expand the methodological basis of reliability inference by incorporating information-theoretic measures into the analysis of system lifetimes. On the inferential side, a Example 6 of residual extropy was proposed for exponential lifetimes. The estimator is computationally simple due to its invariance property, and its performance was assessed through both simulation and real-data analysis. These results demonstrate how residual extropy can serve as a practical tool for reliability inference, complementing related measures such as residual Rényi entropy, Tsallis entropy, and dynamical cumulative residual extropy.

Future research directions include deriving closed-form expressions for broader lifetime families such as Weibull, gamma, and log-logistic, particularly when 2r<n; extending the framework to heterogeneous or dependent components, including copula-based and common-cause models; and developing estimation procedures for censored or truncated data, with rigorous study of asymptotic properties. Exploring semi-parametric, nonparametric, or Bayesian methods may yield more robust inference. Finally, applying residual extropy to system design, optimization, and maintenance scheduling represents a promising avenue for translating theoretical advances into practical reliability solutions.

## Figures and Tables

**Table 1:** Bounds on $R\left(T_{r|n:G};t\right)$ derived from Theorem 4.

Distribution	Probability Density Function	Lower Bound
Half-Cauchy distribution T∼HC(0,1)	$h(x)=\frac{2}{\pi \left(1+x^{2}\right)},x>0$,	$R\left(U_{r|n:G};H(t)\right)\left(\frac{2}{\pi }\right)$
Half-normal distribution T∼HN(μ,σ)	$h(x)=\frac{2}{\sigma \sqrt{2\pi }}e^{-(x-\mu {)}^{2}/2\sigma^{2}},x>\mu >0$,	$R\left(U_{r|n:G};H(t)\right)\left(\frac{2}{\sigma \sqrt{2\pi }}\right)$
Generalized exponential distribution T∼GE(λ,β)	$h(x)=\frac{\lambda }{\beta }e^{-\frac{x}{\beta }}{\left(1-e^{-\frac{x}{\beta }}\right)}^{\lambda -1},x>0$,	$R\left(U_{r|n:G};H(t)\right)\beta {\left(1-\frac{1}{\lambda }\right)}^{1-\lambda }$
Generalized gamma distribution T∼GG(b,c)	$h(x)=\frac{b^{c}}{\Gamma (c)}x^{c-1}e^{-bx},x>0$,	$R\left(U_{r|n:G};H(t)\right)\frac{b(c-1{)}^{c-1}e^{1-c}}{\Gamma (c)}$

**Table 2:** Alternative probability distributions for evaluating the power of the test statistic.

Distribution	PDF	Support	MLE of λ
Exponential	$h(t;\lambda )=\frac{1}{\lambda }e^{-\frac{t}{\lambda }}$	t>0,λ>0	$\frac{\sum_{i=1}^{N}\;\;t_{i}}{N}$
Rayleigh	$h(t;\lambda )=\frac{t}{\lambda^{2}}e^{-{\left(\frac{t}{\sqrt{2}\lambda }\right)}^{2}}$	t>0,λ>0	$\sqrt{\frac{1}{2N}\sum_{i=1}^{N}t_{i}^{2}}$
Lomax	$h(t;\lambda )=\frac{1}{\lambda }{\left(1+t\right)}^{-\left(\frac{1}{\lambda }+1\right)}$	t>0,λ>0	$\frac{\sum_{i=1}^{N}\;\;\log\left(1+t_{i}\right)}{N}$

**Table 3:** Exponential-Based Estimates of $R\left(T_{r|6:G};t\right)$ for Boeing 720 air conditioning equipment.

t	$R\left(T_{3|6:G};t\right)$	$\hat{R}\left(T_{3|6:G};t\right)$	$R\left(T_{4|6:G};t\right)$	$\hat{R}\left(T_{4|6:G};t\right)$	$R\left(T_{5|6:G};t\right)$	$\hat{R}\left(T_{5|6:G};t\right)$	$R\left(T_{6|6:G};t\right)$	$\hat{R}\left(T_{6|6:G};t\right)$
0.1	−0.003534	−0.003077	−0.005502	−0.005169	−0.008624	−0.007460	−0.012369	−0.017131
0.5	−0.003535	−0.003228	−0.005520	−0.005691	−0.008633	−0.009790	−0.012369	−0.011608
0.9	−0.003536	−0.005007	−0.005538	−0.005612	−0.008643	−0.010081	−0.012369	−0.009924
1.2	−0.003538	−0.003808	−0.005551	−0.006283	−0.008650	−0.006111	−0.012369	−0.011671
2.0	−0.003544	−0.004010	−0.005587	−0.005290	−0.008670	−0.010792	−0.012369	−0.013997

## Data Availability

All data generated or analyzed during this study are included in this published article.

## Appendix A

**Table A1:** The bias and RMSE of the estimate of residual extropy of consecutive r-out-of-n:G systems with exponential component lifetimes for different choices of N when λ=0.5.

			N = 20		N = 30		N = 40		N = 50	
n	r	t	Bias	RMSE	Bias	RMSE	Bias	RMSE	Bias	RMSE
5	3	0.1	−0.066224	0.314588	−0.049674	0.245272	−0.033496	0.209265	−0.024395	0.185371
		0.5	−0.085930	0.384733	−0.057343	0.301225	−0.037253	0.261206	−0.033011	0.229065
		0.9	−0.073967	0.388768	−0.052329	0.308288	−0.035647	0.264959	−0.042427	0.233780
		1.2	−0.081779	0.391781	−0.048930	0.310255	−0.036848	0.256642	−0.030364	0.227412
		2.0	−0.076323	0.380605	−0.038799	0.302454	−0.037933	0.248844	−0.033067	0.230345
	4	0.1	−0.090307	0.470968	−0.061544	0.351371	−0.056444	0.301957	−0.036696	0.266503
		0.5	−0.108948	0.516117	−0.068862	0.401021	−0.062220	0.336254	−0.039555	0.301121
		0.9	−0.110930	0.508109	−0.078012	0.401196	−0.060583	0.332997	−0.036571	0.303319
		1.2	−0.107990	0.519740	−0.067249	0.401161	−0.049669	0.349969	−0.037885	0.298688
		2.0	−0.101718	0.528394	−0.073977	0.398898	−0.054977	0.338098	−0.040891	0.298947
	5	0.1	−0.112029	0.640807	−0.082744	0.497024	−0.070229	0.425274	−0.049832	0.378340
		0.5	−0.130957	0.637637	−0.101029	0.494605	−0.057133	0.415593	−0.042438	0.369802
		0.9	−0.135012	0.639473	−0.075179	0.508598	−0.066036	0.418729	−0.046762	0.371878
		1.2	−0.139149	0.610799	−0.087714	0.494106	−0.062906	0.416451	−0.052269	0.362801
		2.0	−0.117708	0.635790	−0.085418	0.503837	−0.059071	0.415535	−0.056513	0.379351

**Table A2:** The bias and RMSE of the estimate of residual extropy of consecutive r-out-of-n:G systems with exponential component lifetimes for different choices of N when λ=1.

			N = 20		N = 30		N = 40		N = 50	
n	r	t	Bias	RMSE	Bias	RMSE	Bias	RMSE	Bias	RMSE
5	3	0.1	−0.035392	0.143554	−0.018093	0.113364	−0.015907	0.092977	−0.012307	0.082294
		0.5	−0.037778	0.188613	−0.026222	0.140229	−0.018028	0.120598	−0.017438	0.106232
		0.9	−0.042676	0.195413	−0.024752	0.151379	−0.018475	0.128439	−0.014729	0.112804
		1.2	−0.037215	0.193026	−0.030440	0.151203	−0.023369	0.133015	−0.015837	0.113601
		2.0	−0.042279	0.193518	−0.026119	0.153052	−0.018581	0.131189	−0.014938	0.115299
	4	0.1	−0.046174	0.225883	−0.031823	0.172738	−0.025749	0.149112	−0.017984	0.129052
		0.5	−0.045975	0.249295	−0.034695	0.194172	−0.027223	0.165976	−0.017536	0.144899
		0.9	−0.053012	0.258403	−0.036383	0.200060	−0.022545	0.169511	−0.021434	0.149731
		1.2	−0.054069	0.258368	−0.035435	0.198919	−0.025591	0.168565	−0.019482	0.148383
		2.0	−0.057473	0.259501	−0.034131	0.200689	−0.026455	0.173329	−0.021231	0.151649
	5	0.1	−0.074305	0.308292	−0.042718	0.253138	−0.028636	0.210196	−0.022517	0.182191
		0.5	−0.076699	0.321788	−0.046878	0.247679	−0.032379	0.213634	−0.027253	0.184054
		0.9	−0.063367	0.321546	−0.051576	0.246120	−0.029813	0.205894	−0.026457	0.190405
		1.2	−0.059447	0.318281	−0.041618	0.253140	−0.031824	0.215008	−0.028468	0.187964
		2.0	−0.059360	0.315454	−0.043770	0.247269	−0.032113	0.211321	−0.021402	0.188700

**Table A3:** The bias and RMSE of the estimate of residual extropy of consecutive r-out-of-n:G systems with exponential component lifetimes for different choices of N when λ=2.

			N = 20		N = 30		N = 40		N = 50	
n	r	t	Bias	RMSE	Bias	RMSE	Bias	RMSE	Bias	RMSE
5	3	0.1	−0.067643	0.323217	−0.039620	0.250601	−0.035277	0.203316	−0.028151	0.183584
		0.5	−0.087951	0.393439	−0.047189	0.306124	−0.037493	0.258973	−0.032059	0.222218
		0.9	−0.071776	0.393429	−0.057114	0.310429	−0.044539	0.256037	−0.031417	0.229323
		1.2	−0.072666	0.384819	−0.048050	0.300470	−0.041600	0.254391	−0.032625	0.230711
		2.0	−0.083938	0.388912	−0.057015	0.305312	−0.038061	0.252038	−0.030249	0.224856
	4	0.1	−0.110652	0.459938	−0.063464	0.371098	−0.050267	0.309822	−0.034252	0.272512
		0.5	−0.097647	0.510737	−0.070674	0.398359	−0.052961	0.334673	−0.040356	0.298153
		0.9	−0.115989	0.517071	−0.066843	0.408897	−0.051785	0.339806	−0.054132	0.314601
		1.2	−0.097652	0.514063	−0.068308	0.400150	−0.052447	0.342001	−0.046807	0.296733
		2.0	−0.109741	0.506129	−0.076286	0.401245	−0.050164	0.343238	−0.040773	0.300634
	5	0.1	−0.127115	0.642847	−0.096041	0.514725	−0.052853	0.429019	−0.042172	0.370054
		0.5	−0.120246	0.632471	−0.087842	0.492236	−0.061360	0.413476	−0.050568	0.371091
		0.9	−0.123977	0.619784	−0.076744	0.494405	−0.056177	0.424798	−0.052135	0.371922
		1.2	−0.120518	0.639902	−0.084978	0.496025	−0.064111	0.425761	−0.047310	0.374579
		2.0	−0.144005	0.633773	−0.086496	0.500675	−0.062268	0.412549	−0.049757	0.375764

**Table A4:** The bias and RMSE of the estimate of residual extropy of consecutive r-out-of-n:G systems with Rayleigh component lifetimes for different choices of N when λ=0.5.

			N = 20		N = 30		N = 40		N = 50	
n	r	t	Bias	RMSE	Bias	RMSE	Bias	RMSE	Bias	RMSE
5	3	0.1	−0.019410	0.094962	−0.009489	0.074706	−0.008239	0.064397	−0.006864	0.056967
		0.5	−0.071171	0.369722	−0.050968	0.291289	−0.038749	0.249405	−0.027490	0.214832
		0.9	−0.135766	0.725580	−0.104440	0.546468	−0.083944	0.464267	−0.063286	0.420929
		1.2	−0.189058	0.912255	−0.127313	0.729511	−0.094175	0.610606	−0.065195	0.543931
		2.0	−0.336754	1.545538	−0.201810	1.198421	−0.129687	1.002721	−0.147198	0.894646
	4	0.1	−0.020068	0.127285	−0.014508	0.101236	−0.013768	0.085888	−0.010673	0.078653
		0.5	−0.020815	0.503678	−0.070903	0.391577	−0.051315	0.334926	−0.033836	0.300815
		0.9	−0.099184	0.928263	−0.124615	0.718292	−0.082456	0.614117	−0.077519	0.544725
		1.2	−0.209940	1.239788	−0.150353	0.962747	−0.122382	0.804038	−0.105740	0.713534
		2.0	−0.241525	2.035972	−0.273328	1.593777	−0.217311	1.362852	−0.132819	1.195953
	5	0.1	−0.032932	0.174165	−0.018696	0.134441	−0.018441	0.119329	−0.011909	0.102262
		0.5	−0.129979	0.641140	−0.075214	0.509492	−0.073035	0.420691	−0.050605	0.369933
		0.9	−0.242783	1.119166	−0.145670	0.881479	−0.126703	0.763648	−0.108333	0.664930
		1.2	−0.319493	1.538127	−0.200321	1.201260	−0.141429	1.003724	−0.145807	0.890896
		2.0	−0.507347	2.558486	−0.326551	2.012387	−0.242880	1.682465	−0.173647	1.490342

**Table A5:** The bias and RMSE of the estimate of residual extropy of consecutive r-out-of-n:G systems with Rayleigh component lifetimes for different choices of N when λ=1.

			N = 20		N = 30		N = 40		N = 50	
n	r	t	Bias	RMSE	Bias	RMSE	Bias	RMSE	Bias	RMSE
5	3	0.1	−0.005665	0.042861	−0.004110	0.035075	−0.003181	0.030013	−0.003042	0.026393
		0.5	−0.017609	0.084430	−0.012114	0.064272	−0.008190	0.054668	−0.006358	0.048256
		0.9	−0.037292	0.166653	−0.021084	0.125268	−0.018035	0.107016	−0.015445	0.096302
		1.2	−0.047958	0.231233	−0.034973	0.182926	−0.022896	0.148637	−0.015360	0.135868
		2.0	−0.079369	0.389244	−0.056936	0.309403	−0.041696	0.262003	−0.033413	0.231260
	4	0.1	−0.008422	0.052928	−0.005597	0.042163	−0.003709	0.035702	−0.003430	0.032054
		0.5	−0.022939	0.119537	−0.013495	0.094757	−0.012512	0.079816	−0.008376	0.070122
		0.9	−0.051982	0.221947	−0.032621	0.176321	−0.026229	0.150267	−0.017808	0.129317
		1.2	−0.059551	0.304675	−0.041278	0.235028	−0.032081	0.207836	−0.026056	0.179176
		2.0	−0.112876	0.512895	−0.065190	0.412052	−0.044531	0.343136	−0.046913	0.305332
	5	0.1	−0.011556	0.067641	−0.007625	0.053517	−0.004724	0.045312	−0.004515	0.040534
		0.5	−0.035174	0.167632	−0.021562	0.127895	−0.015367	0.110098	−0.009162	0.096350
		0.9	−0.054328	0.292367	−0.037807	0.227515	−0.025836	0.190176	−0.025953	0.169310
		1.2	−0.064270	0.373451	−0.057461	0.299810	−0.036819	0.253103	−0.032386	0.222476
		2.0	−0.123340	0.645224	−0.081259	0.496744	−0.062627	0.420577	−0.050880	0.380345

**Table A6:** The bias and RMSE of the estimate of residual extropy of consecutive r-out-of-n:G systems with Rayleigh component lifetimes for different choices of N when λ=2.

			N = 20		N = 30		N = 40		N = 50	
n	r	t	Bias	RMSE	Bias	RMSE	Bias	RMSE	Bias	RMSE
5	3	0.1	−0.003622	0.021230	−0.002580	0.016585	−0.001548	0.014498	−0.001348	0.012946
		0.5	−0.004238	0.025672	−0.002720	0.019903	−0.002151	0.017453	−0.001570	0.015354
		0.9	−0.007431	0.036818	−0.004670	0.028966	−0.003590	0.024394	−0.002868	0.021250
		1.2	−0.009602	0.049212	−0.007181	0.039460	−0.005190	0.032272	−0.004004	0.029054
		2.0	−0.022306	0.092095	−0.013256	0.072803	−0.009489	0.060873	−0.007266	0.054001
	4	0.1	−0.003911	0.024931	−0.002896	0.019926	−0.002029	0.017113	−0.001701	0.015161
		0.5	−0.006649	0.034752	−0.003769	0.028269	−0.003451	0.024114	−0.002614	0.021603
		0.9	−0.011623	0.054840	−0.007064	0.042740	−0.005280	0.036418	−0.004699	0.031863
		1.2	−0.016417	0.070310	−0.007678	0.056308	−0.008278	0.047212	−0.006372	0.041533
		2.0	−0.027405	0.125927	−0.017900	0.096887	−0.011717	0.083224	−0.009716	0.074708
	5	0.1	−0.005235	0.030703	−0.003809	0.024555	−0.002657	0.020983	−0.001866	0.018566
		0.5	−0.009379	0.048841	−0.005705	0.039470	−0.004447	0.032340	−0.003635	0.028458
		0.9	−0.015715	0.077014	−0.009637	0.059305	−0.007292	0.049851	−0.005995	0.043912
		1.2	−0.019403	0.096377	−0.015500	0.077503	−0.010138	0.065596	−0.007126	0.057571
		2.0	−0.032098	0.157088	−0.022488	0.126203	−0.014875	0.107313	−0.014721	0.092944

**Table A7:** The bias and RMSE of the estimate of residual extropy of consecutive r-out-of-n:G systems with Lomax component lifetimes for different choices of N when λ=0.5.

			N = 20		N = 30		N = 40		N = 50	
n	r	t	Bias	RMSE	Bias	RMSE	Bias	RMSE	Bias	RMSE
5	3	0.1	−0.067248	0.294395	−0.041347	0.221712	−0.029447	0.187684	−0.027490	0.164239
		0.5	−0.056641	0.253432	−0.036821	0.199113	−0.024835	0.167111	−0.017540	0.149702
		0.9	−0.040584	0.212374	−0.029988	0.161356	−0.021370	0.137358	−0.017643	0.120668
		1.2	−0.034574	0.177771	−0.025639	0.136038	−0.016920	0.116409	−0.014216	0.104595
		2.0	−0.024095	0.132672	−0.017307	0.099271	−0.014464	0.085307	−0.009488	0.076012
	4	0.1	−0.090733	0.412259	−0.061121	0.325951	−0.042168	0.274629	−0.037792	0.248682
		0.5	−0.073091	0.335889	−0.051848	0.262351	−0.036057	0.223103	−0.030340	0.198583
		0.9	−0.055394	0.271062	−0.038214	0.214473	−0.027829	0.179778	−0.020382	0.160412
		1.2	−0.046523	0.238672	−0.036037	0.182016	−0.026322	0.157121	−0.022467	0.138449
		2.0	−0.033471	0.174377	−0.026497	0.134515	−0.018884	0.112687	−0.011666	0.100422
	5	0.1	−0.119858	0.579641	−0.072157	0.452838	−0.053816	0.380682	−0.044691	0.338109
		0.5	−0.082804	0.420643	−0.061134	0.330123	−0.042121	0.281171	−0.033016	0.246984
		0.9	−0.067961	0.332135	−0.048821	0.264733	−0.038716	0.223500	−0.025240	0.193100
		1.2	−0.060411	0.286903	−0.038704	0.230930	−0.027902	0.191775	−0.019882	0.167827
		2.0	−0.044871	0.213451	−0.026254	0.170397	−0.025392	0.136542	−0.015234	0.125590

**Table A8:** The bias and RMSE of the estimate of residual extropy of consecutive r-out-of-n:G systems with Lomax component lifetimes for different choices of N when λ=1.

			N = 20		N = 30		N = 40		N = 50	
n	r	t	Bias	RMSE	Bias	RMSE	Bias	RMSE	Bias	RMSE
5	3	0.1	−0.030578	0.124778	−0.016568	0.098240	−0.013040	0.083668	−0.010560	0.070728
		0.5	−0.028006	0.118305	−0.015811	0.091737	−0.010718	0.077196	−0.011597	0.068715
		0.9	−0.021517	0.095490	−0.014606	0.074787	−0.010621	0.065076	−0.008412	0.056771
		1.2	−0.021193	0.087625	−0.011162	0.068550	−0.009332	0.056787	−0.007454	0.049781
		2.0	−0.015668	0.064168	−0.008425	0.051392	−0.006754	0.043040	−0.005253	0.037356
	4	0.1	−0.039880	0.196610	−0.025180	0.152852	−0.021131	0.131535	−0.016428	0.117008
		0.5	−0.036657	0.161604	−0.022758	0.127433	−0.015379	0.108384	−0.013737	0.094184
		0.9	−0.027412	0.130078	−0.019510	0.100833	−0.013846	0.086120	−0.011185	0.076118
		1.2	−0.025199	0.114838	−0.014536	0.090479	−0.011839	0.075418	−0.008269	0.067068
		2.0	−0.016184	0.084610	−0.010761	0.065488	−0.008002	0.055917	−0.007899	0.050378
	5	0.1	−0.058387	0.285605	−0.040062	0.227131	−0.029312	0.186988	−0.022116	0.167602
		0.5	−0.043571	0.205410	−0.029244	0.158389	−0.021236	0.142681	−0.014997	0.124184
		0.9	−0.035562	0.163942	−0.020296	0.129459	−0.018733	0.110056	−0.012726	0.098319
		1.2	−0.028554	0.140806	−0.019528	0.111962	−0.014573	0.096013	−0.011942	0.083613
		2.0	−0.022076	0.106398	−0.014144	0.084199	−0.008539	0.067995	−0.006439	0.061413

**Table A9:** The bias and RMSE of the estimate of residual extropy of consecutive r-out-of-n:G systems with Lomax component lifetimes for different choices of N when λ=2.

			N = 20		N = 30		N = 40		N = 50	
n	r	t	Bias	RMSE	Bias	RMSE	Bias	RMSE	Bias	RMSE
5	3	0.1	−0.012139	0.053541	−0.007906	0.040293	−0.005707	0.034482	−0.004826	0.030210
		0.5	−0.011609	0.048795	−0.007495	0.037624	−0.005928	0.031894	−0.004107	0.028014
		0.9	−0.009708	0.041446	−0.006766	0.032777	−0.005015	0.027753	−0.003909	0.024238
		1.2	−0.009262	0.037895	−0.005976	0.029184	−0.004325	0.024869	−0.003305	0.021692
		2.0	−0.006238	0.030350	−0.004366	0.022748	−0.003702	0.019104	−0.002346	0.016770
	4	0.1	−0.020331	0.094116	−0.013100	0.071392	−0.011219	0.060617	−0.007824	0.053913
		0.5	−0.014894	0.075501	−0.012018	0.057369	−0.007755	0.049040	−0.005077	0.042589
		0.9	−0.013580	0.061341	−0.009344	0.047664	−0.007003	0.039182	−0.005761	0.035730
		1.2	−0.011172	0.053683	−0.007143	0.041875	−0.005554	0.034728	−0.004706	0.031128
		2.0	−0.009162	0.039295	−0.006187	0.032297	−0.003435	0.026794	−0.003441	0.023458
	5	0.1	−0.026003	0.143788	−0.020103	0.110235	−0.016765	0.093530	−0.011435	0.081519
		0.5	−0.020475	0.103874	−0.012951	0.078091	−0.012151	0.068575	−0.008017	0.059602
		0.9	−0.016637	0.083201	−0.011091	0.062676	−0.007415	0.053903	−0.007835	0.047979
		1.2	−0.015221	0.069844	−0.009548	0.054906	−0.005861	0.045959	−0.005324	0.041565
		2.0	−0.010398	0.052263	−0.007010	0.039623	−0.006447	0.034103	−0.004370	0.030318
